# Molecular mechanism of LIP05 derived from *Monascus purpureus* YJX-8 for synthesizing fatty acid ethyl esters under aqueous phase

**DOI:** 10.3389/fmicb.2022.1107104

**Published:** 2023-01-12

**Authors:** Jingrong Zhao, Youqiang Xu, Hongyun Lu, Dong Zhao, Jia Zheng, Mengwei Lin, Xin Liang, Ze Ding, Wenqi Dong, Maochen Yang, Weiwei Li, Chengnan Zhang, Baoguo Sun, Xiuting Li

**Affiliations:** ^1^Key Laboratory of Brewing Microbiome and Enzymatic Molecular Engineering, China General Chamber of Commerce, Beijing Technology and Business University, Beijing, China; ^2^Beijing Advanced Innovation Center for Food Nutrition and Human Health, Beijing Technology and Business University, Beijing, China; ^3^Wuliangye Yibin Co., Ltd., Yibin, Sichuan, China; ^4^School of Food and Health, Beijing Technology and Business University (BTBU), Beijing, China

**Keywords:** strong-flavor Baijiu, fatty acid ethyl ester, enzymatic esterification, aqueous phase, molecular mechanism

## Abstract

Fatty acid ethyl esters are important flavor chemicals in strong-flavor Baijiu. *Monascus purpureus* YJX-8 is recognized as an important microorganism for ester synthesis in the fermentation process. Enzyme LIP05 from YJX-8 can efficiently catalyze the synthesis of fatty acid ethyl esters under aqueous phase, but the key catalytic sites affecting esterification were unclear. The present work combined homology modeling, molecular dynamics simulation, molecular docking and site-directed mutation to analyze the catalytic mechanism of LIP05. Protein structure modeling indicated LIP05 belonged to α/β fold hydrolase, contained a lid domain and a core catalytic pocket with conserved catalytic triad Ser150-His215-Asp202, and the oxyanion hole composed of Gly73 and Thr74. Ile30 and Leu37 of the lid domain were found to affect substrate specificity. The π-bond stacking between Tyr116 and Tyr149 played an important role in stabilizing the catalytic active center of LIP05. Tyr116 and Ile204 determined the substrate spectrum by composing the substrate-entrance channel. Residues Leu83, Ile204, Ile211 and Leu216 were involved in forming the hydrophobic substrate-binding pocket through steric hindrance and hydrophobic interaction. The catalytic mechanism for esterification in aqueous phase of LIP05 was proposed and provided a reference for clarifying the synthesis of fatty acid ethyl esters during the fermentation process of strong-flavor Baijiu.

## Introduction

Baijiu is one of the world’s six most famous distilled beverages and an important component of traditional Chinese fermented food (Jin et al., 2017; Liu and Sun, 2018). There are 12 types of baijiu with different flavor characteristics, among which strong-flavor baijiu accounts for more than 70% of the baijiu market share (Huang et al., 2022). However, the quality improvement of strong-flavor baijiu is limited since the manufacturing process is still based on an empirical mode with low efficiency and poor stability between batches (Xu et al., 2022b). The reason is the core functional microorganisms and the formation mechanism of flavor chemicals are still not clear (Wu et al., 2021).

Esters are important flavor chemicals in baijiu, and 510 kinds of esters are reported until now (Hong et al., 2020). Ethyl esters are the compounds with high concentrations and great flavor contributions in baijiu (Xu et al., 2022b). Ethyl hexanoate is reported as the principal flavor chemical in strong-flavor baijiu with a smell similar to the mixed smell of pineapple, mud, sauerkraut, and plant ash, and is recognized as a characteristic of high quality product (Wei et al., 2020). In addition, fatty acid ethyl esters such as ethyl butyrate, ethyl valerate, and ethyl octanoate are also contributed significantly to the flavor of product, and these esters together form the unique flavor of strong-flavor baijiu (Hong et al., 2021; Xu et al., 2022b).

Flavor chemicals were synthesized by a large number of microorganisms during the fermentation process of baijiu (Du et al., 2021). It is worth noting that although lots of microorganisms have been found in the fermentation process, the core functional microorganisms and how to regulate the microbes for synthesizing the desired flavor compounds such as ethyl esters are still unclear (Wu et al., 2021). Although some studies have reported that bacteria, mold, and yeast show abilities to synthesize esters, the formation mechanism is still unrevealed (Wu et al., 2021). The enzymes produced by microbes are the crucial functional elements to catalyze the synthesis of ethyl esters. There are four main pathways for microbial ester synthesis (Kruis et al., 2019), the first pathway uses alcohols and aldehydes as substrates for hemiacetal dehydrogenation to generate esters. The second pathway catalyzes the synthesis of esters using ketones as substrate and NAD (P) H as the cofactor by Baeyer-Villiger monooxygenases. The third pathway is the synthesis of ethyl acetate catalyzed by alcohol acyltransferases using alcohol and acyl-CoA as the substrates. The fourth pathway is esterification reaction catalyzed by ester synthase using alcohols and acids as the substrates, and is usually recognized as the main pathway to synthesize fatty acid ethyl esters in strong-flavor baijiu (Xu et al., 2022a). Therefore, the mechanism investigation of enzymatic esterification is a key step for revealing the formation mechanism of esters in Baijiu fermentation process.

Most of enzymes with the ability to synthesize ethyl esters belong to the α/β fold hydrolases, such as esterase, lipase and cutinase, which are mainly produced by microorganisms in the fermentation process (Feng et al., 2021; Tu et al., 2022). In general, the α/β fold hydrolases consists of two domains, a core catalytic domain and a lid domain. The catalytic domain contains the catalytic triad Ser-His-Asp/Glu and the substrate binding sites (Rauwerdink and Kazlauskas, 2015). The catalytic active center is covered by a lid domain, which prevents the substrate entrance directly (Khan et al., 2017). To perform the catalytic function of esterification, the enzyme needs to undergo a conformational change to open the lid domain and expose the active center to allow the substrate entrance into the catalytic active center, thus achieving catalysis. Esterification is usually carried out under organic phase, while during the fermentation process of baijiu, the water content of fermented grains is about 53–58%, and generally recognized as an aqueous phase (Xu et al., 2017). In aqueous phase, ester hydrolysis reactions often occur and lead to the instability of ester synthesis during the fermentation process of baijiu (Xu et al., 2022a). Although there are investigations about the organic reactions successfully performed in an aqueous phase (Li and Chen, 2006; Simon and Li, 2012; Kitanosono et al., 2018), few studies reported the acid-alcohol esterification under aqueous phase. Therefore, explaining the mechanism of the enzyme to catalyze the esters synthesis in aqueous phase will give us an in-depth understanding of the synthetic mechanism of esters in baijiu fermentation process.

In our previous work, an enzyme named LIP05 was identified for ester synthesis in aqueous phase from *Monascus purpureus* YJX-8 (Xu et al., 2021). The enzyme responsible for catalyzing ester synthesis in the aqueous phase was rarely reported and the mechanism was unclear. In this work, based on homology modeling, molecular dynamics simulation, molecular docking and site-directed mutation, the key amino acid residues were analyzed and the catalytic mechanism of LIP05 in aqueous phase was proposed. This will help to reveal the molecular mechanism of *M. purpureus* for ester synthesis in aqueous phase, and promote the application of the strain and enzyme resources for baijiu fermentation.

## Materials and methods

### Materials and media

The strain *Monascus purpureus* YJX-8 was isolated from the fermentation starter of Baijiu in our previous work (Xu et al., 2021). LIP05 was identified as the key enzyme of ester synthesis in aqueous phase and encoded by the gene numbered GME7409. *Escherichia coli* BL21 (DE3) were used as the host for heterologous expression of LIP05. Butyric acid, pentanoic acid, hexanoic acid, octanoic acid, decanoic acid, ethyl butyrate, ethyl pentanoate, ethyl hexanoate, ethyl octanoate, and ethyl decanoate standards were purchased from Sigma Aldrich (St Louis, MO). Luria Bertani (LB) medium (10 g/l tryptone, 5 g/l yeast extract, 10 g/l NaCl) was used to cultivate the recombinant strains. Agarose (2%) was added to generate the solid LB medium. All other chemicals were analytical grade reagents and commercially available.

### Homologous modeling and molecular docking of LIP05

Template 4PSC from *Trichoderma reesei* (sequence identity of 46.40% and query coverage of 92.82%) was selected as the reference for homologous modeling (Roussel et al., 2014). Discovery Studio (V2019) was used to build the 3D protein model evaluated by Ramachandran plot. Molecular docking of LIP05 with butyric acid, pentanoic acid, hexanoic acid, octanoic acid, decanoic acid, ethyl butyrate, ethyl pentanoate, ethyl hexanoate, ethyl octanoate, and ethyl decanoate was performed using AutoDock software (Goodsell et al., 1996). The structures of these small molecule compounds were downloaded from the ZINC database^1^ and defined as the ligands. LIP05 was defined as the receptor. The input file of the receptor and ligands in PDB format were converted into the AutoDock-compatible PDBQT format using MGLTools. The active site of LIP05 was selected as the docking region. The grid center was set at the O^γ^ atom of the catalytic residue Ser150. The conformation with the lowest affinity energy was selected as the most probable binding conformation and visualized by PyMOL.

### Molecular dynamics simulation of LIP05

Molecular dynamics (MD) simulation of the LIP05 was performed using GROMACS version 2022^2^ with the OPLS-AA force field (Kaminski et al., 2001). PDBFIXER^3^ was used to structure preparation of LIP05, added missing amino acids and side chain atoms and formed a protonated state of charged amino acids. Ions with opposite charges were added to neutralize the charge of the system. Additional Na^+^ and Cl^+^ were added to create a salt concentration of 150 mM to simulate the actual experiments. The steepest descent method was used to minimize the energy of the above constructed system to eliminate spatial conflicts. The system was equilibrated at 300 K for 100 ps in the constant temperature and volume. Afterwards, the system was equilibrated at 1.0 bar for 100 ps in the constant pressure and temperature (Páll et al., 2014; Abraham et al., 2015). MD simulation was running for 100 ns. The LINCS algorithm was used to impose rigid constraints on the hydrogen bonds (Hess, 2008a). Electrostatic interactions were treated using the particle-mesh Ewald method (Darden et al., 1993). The Berendsen thermostat (Berendsen et al., 1984) and Parrinello-Rahman barostat (Parrinello and Rahman, 1981) were used to maintain the temperature and pressure. The coordinates and energy were saved every 1.0 ps. The result was analyzed by GROMACS tools (Van Der Spoel et al., 2005; Hess et al., 2008b; Pronk et al., 2013).

### Construction of LIP05 mutants

The expression vector pCold-TF was amplified using the primer pair pCold-TF.f/ pCold-TF.r through polymerase chain reaction (PCR) to generate the linearized vector (Supplementary Table S1). The gene encoding LIP05 was added with the 18 or 20 bp homologous recombinant sequence region by PCR using the respective primer pairs to generate the DNA fragment (Supplementary Table S1). The fragment was integrated with linearized vector DNA to produce the expression vector according to the protocol of the Vazyme ClonExpress II One Step Cloning Kit (Vazyme Biotech, Nanjing, China). The constructed vectors were transferred into the host cell *E. coli* BL21 (DE3) to generate the respective transformants. Using the expression vector carried the gene encoding LIP05 as a template, mutants were constructed by whole plasmid PCR using the respective designed primer pairs as shown in Supplementary Table S1, and transferred into *E. coli* BL21 (DE3) to produce the respective transformants.

### Enzyme expression and purification

The recombinant *E. coli* strains were inoculated into LB medium containing 100 μg/ml Ampicillin sodium and kept at 37°C with stirring at 200 r/min for about 3 h until the OD_600_ value reached 0.6–0.8. Isopropyl β-D-1-thiogalactopyranoside (IPTG) was added and then recombinant *E. coli* strains were kept at 16°C and 200 r/min for 20 h. The cells were collected by centrifugation at 4°C and 8,000 × *g* for 5 min, and were washed twice with Tris–HCl buffer (50 mM, pH 7.5). The cells were disrupted using an ultrasonic cell disrupter and were centrifuged at 4°C and 9,000 × *g* for 20 min to obtain the crude enzyme solution, which was used for purification and esterification in aqueous phase.

The crude enzyme was purified on a Ni Sepharose HP column (1 ml, GE, Uppsala, Sweden) using ÄKTA Fast Protein Liquid Chromatography purification system (GE, Uppsala, Sweden) with phosphate buffer (50 mM, pH 7.4) containing 400 mM NaCl and different concentrations of imidazole. The purified enzyme band was verified by SDS-PAGE, the protein concentration was determined using a BCA protein assay kit (Thermo Fischer Scientific Inc., Rockford, United States).

### Determination of ester synthesis ability under aqueous phase

The aqueous phase reaction system contained the enzyme solution (1 ml), the organic acids of butyric acid, pentanoic acid, hexanoic acid, octanoic acid, decanoic acid (a final concentration of 10 mM for each chemical), and ethanol (a final concentration of 1 M) in citric acid buffer (50 mM, pH 4.0) to a final volume of 10 ml. The reaction system was shaken in a water bath at 37°C with stirring at 150 r/min for about 24 h. Thereafter, 3 ml of n-hexane (with internal standard ethyl oleate added) was added into the reaction system, vortexed for 30 s, and centrifuged at 4°C and 12,000 × *g* for 5 min. The upper layer was filtered and used for qualitative detection of esters by gas chromatography (GC).

### Analytical method

The ester concentration was determined by GC (Agilent 7890B, Santa Clara, CA) using a 19,091 N-213I column (30 m × 0.32 mm × 0.50 μm, Agilent, Santa Clara, CA). The column temperature was kept at 40°C for 5 min, increased to 170°C at 8°C/min, maintained for 10 min, and increased to 240°C at 8°C/min, maintained for 5 min. The carrier gas was nitrogen with a flow rate of 1.0 ml/min. The instrument was equipped with an autosampler and a flame ionization detector. The injection volume was 1.0 μl. Origin 8.0 software was used for statistical analysis. The sequences were aligned using the Clustal algorithm^4^, and the results were visualized using ENDscript/ESPript.^5^

## Results and discussion

### Structure analysis, molecular dynamics simulation and molecular docking of LIP05

**Figure 1:**
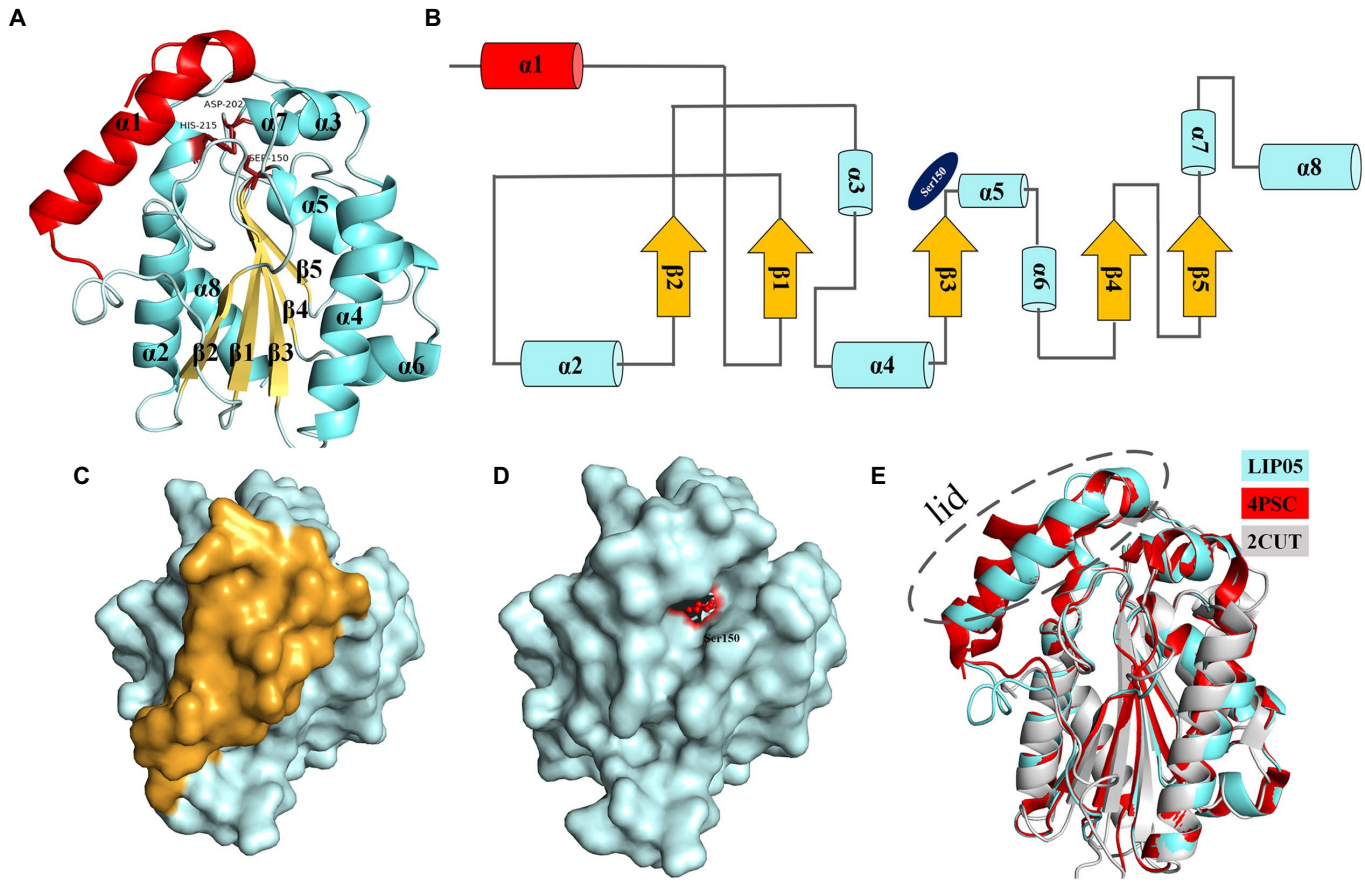
Structure analysis of LIP05. **(A)** Homologous modeling of LIP05. **(B)** Topological structure of LIP05. **(C)** Surface model of LIP05, and yellow color domain was the lid domain. **(D)** Lid domain removed of LIP05, and red color domain was Ser150. **(E)** Structural comparison of LIP05 with enzymes 4PSC and 2CUT.

Ester synthesis reactions catalyzed by α/β fold hydrolase are mostly occurred in organic phase, and few reports focus on enzymatic ester synthesis in aqueous phase (Xu et al., 2021). LIP05 could catalyze the synthesis of fatty acid ethyl esters in aqueous phase, but the catalytic mechanism was still unclear. LIP05 had two domains and belonged to α/β fold hydrolase, the core catalytic domain consisted of 5 β-sheets [β1 (Tyr66-Ala71), β2 (Leu101-Gly105), β3 (His143-Tyr149), β4 (Ser173-Phe177), and β5 (Val194-Ala197)] surrounded by 7 α-helices [α2 (Gly85-Val96), α3 (Ile113-Ala118), α4 (Asp121-Thr138), α5 (Gln151-Lys162), α6 (Ala165-Trp170), α7 (Asn203-Gln206), and α8 (Pro213-Ala232)], the lid domain consisted of 1 α-helix [α1 (Ile30-Thr48); Figures 1A,B]. The catalytic triad was Ser150-His215-Asp202 (Supplementary Figure S1), and belonged to the classical catalytic triad of α/β fold hydrolase (Rauwerdink and Kazlauskas, 2015; Zong et al., 2022). Ser150 was located on the “nucleophile elbow” connecting β3 and α5, His215 was located on the α8, and Asp202 was located on the loop between β5 and α7 (Figures 1A,B). The catalytic active center was covered by the lid domain (Figures 1C,D). The enzyme activity of the LIP05-50 (lid-removed mutant) was increased by 52.50% ± 18.90%, but the stability was decreased, indicating that the lid domain played an important role in maintaining the stability of the enzyme, and the removing of the lid domain was conducive to the improvement of enzyme activity (Xu et al., 2021). The enzymes 4PSC and 2CUT had similar structure with that of LIP05 (Figure 1E). 4PSC had 5 β-sheets surrounded by 8 α-helices, and the lid domain consisted of 2 α-helices (α1 and α2). Similarly, both LIP05 and 4PSC showed high enzyme activity in an acidic environment, which might be due to the strong interaction of the oil–water interface in a low pH environment and beneficial to the opening of the lid domain (Roussel et al., 2014). On the contrary, 2CUT had no lid domain, and the active center was exposed to the environment, and therefore, there was no interface activation phenomenon (Martinez et al., 1994).

**Figure 2:**
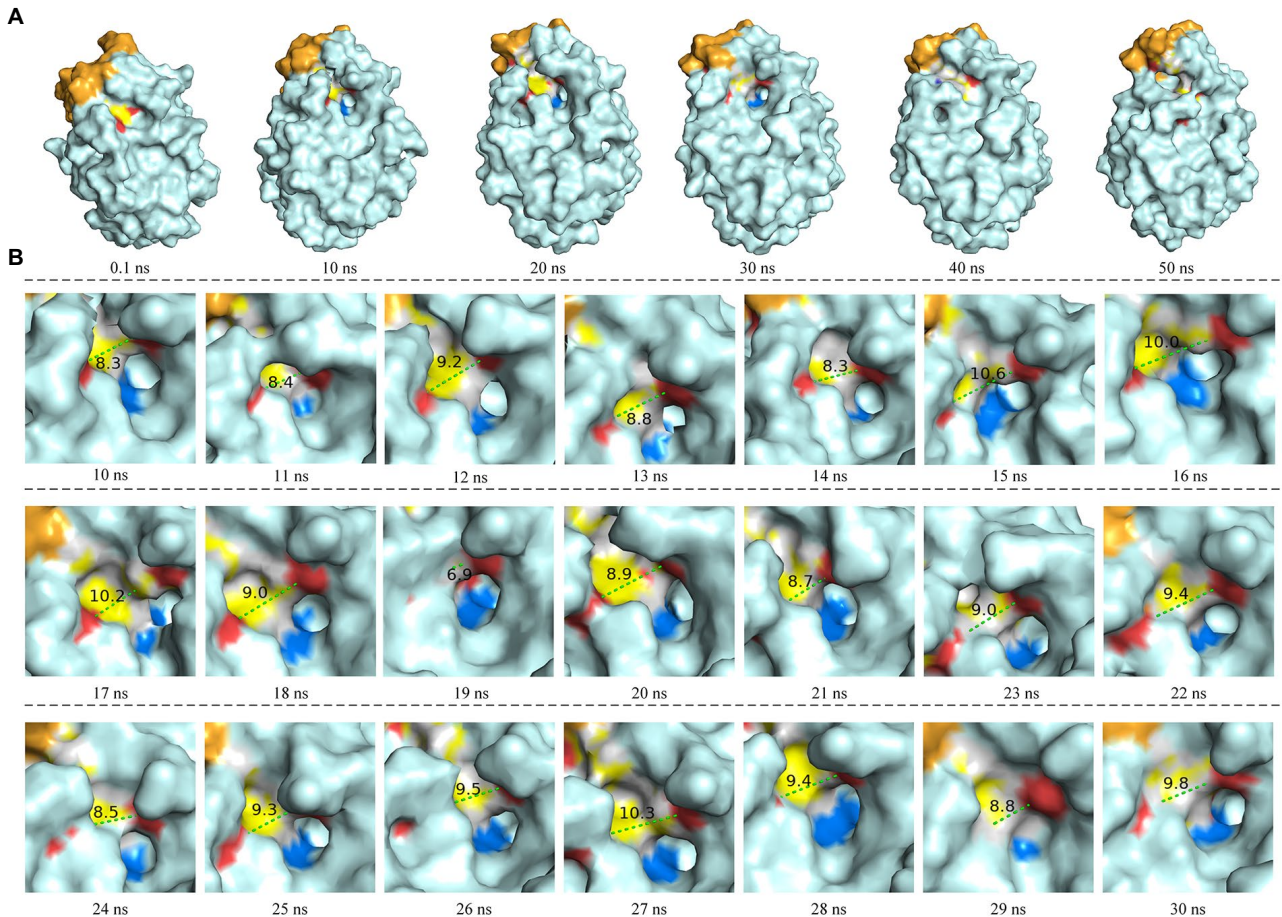
Molecular dynamics simulation of LIP05. **(A)** Motion trajectory from 0.1 ns to 50 ns. **(B)** The distance of carboxy oxygen atoms between Tyr116 and Ile204 from 10 ns to 30 ns. The blue color domain was the active center Ser150.

The molecular dynamics simulation of LIP05 in aqueous phase was analyzed. The RMSD of the protein increased from 0.2 nm to 0.35 nm and finally stabilized at 0.3 nm (Supplementary Figure S2). The radius of gyration was increased significantly from 10 ns to 20 ns (Supplementary Figure S3), indicating that the tightness of protein folding was decreased, and the looseness of protein was increased. Furthermore, the solvent accessible surface area (SASA) also suggested that the hydrophobic area inside the protein was exposed by the opening of the lid domain from 10 ns to 30 ns (Supplementary Figure S4). At this time, the active center was exposed because of the opening of the lid domain, and it was covered again after 40 ns (Figure 2A). It was discovered that the distance between the Tyr116 and Ile204 played a critical role in the exposure of the active center, which determined the substrate spectrum by composing the substrate-entrance channel. The distance of carboxy oxygen atoms between Tyr116 and Ile204 from 10 ns to 30 ns was fluctuated between 6.90 Å – 10.60 Å (Figure 2B), which could accommodate the substrate entrance into the catalytic active pocket, and explained why LIP05 could catalyze the synthesis of fatty acid ethyl esters in aqueous phase. However, the substrate interacted with enzyme after entered the catalytic active pocket was still unclear. Molecular docking was used to predict the interactions between enzyme and substrates (butyric acid, pentanoic acid, hexanoic acid, octanoic acid, and decanoic acid) and products (ethyl butyrate, ethyl pentanoate, ethyl hexanoate, ethyl octanoate, and ethyl decanoate; Supplementary Figure S5). All the docking results were summarized in Supplementary Table S2. Docking with hexanoic acid indicated that LIP05 could interact with the ligand by hydrogen bond through the Thr74 (2.60 Å; Supplementary Figure S5), and LIP05 could interact with the ligand ethyl hexanoate by hydrogen bonds through the Ser150 (2.60 Å) and His215 (2.20 Å; Supplementary Figure S5). In addition, the hydrophobic interaction was also a key force between the enzyme and the substrates. LIP05 could interact with substrates by hydrophobic interaction through the residues Ile30, Leu37, Gly73, Leu83, Tyr116, Tyr149, Ile204, Ile211 and Leu216 (Supplementary Figure S6).

### Construction of LIP05 mutants

Molecular docking indicated LIP05 could form hydrogen bonds and hydrophobic interactions with substrates under 10 residues (Ile30, Leu37, Gly73, Thr74, Leu83, Tyr116, Tyr149, Ile204, Ile211, and Leu216). Hydrogen bonds and hydrophobic interactions could determine protein folding and played an important role in maintaining protein structure (Brandl et al., 2001). In order to reveal how these residues affected the ester catalysis of LIP05, all these residues were mutated by different types of amino acids according to steric hindrance, hydrophilicity and hydrophobicity of the amino acids to explore the catalytic mechanism of LIP05 (Supplementary Figures S7, S8).

### Determination of ester synthesis ability of LIP05 mutants under aqueous phase

#### Key amino acid residues in the lid domain

**Figure 3:**
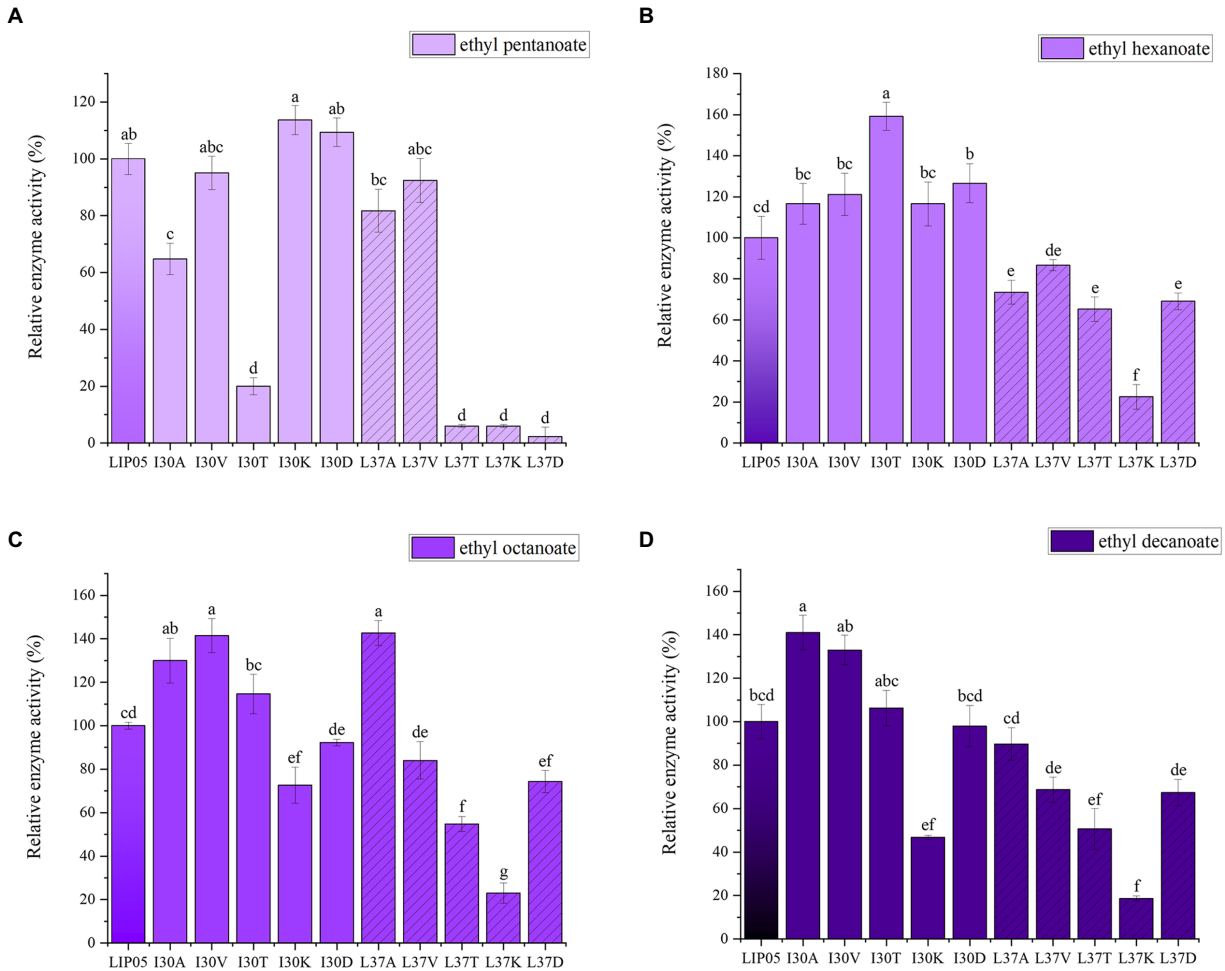
The ester synthesis ability of the mutants at Ile30 and Leu37. **(A)** Synthesis of ethyl pentanoate, **(B)** Synthesis of ethyl hexanoate, **(C)** Synthesis of ethyl octanoate, **(D)** Synthesis of ethyl decanoate. Bars with different letters (a-d) are significantly different (*p*<0.05).

The most suitable substrates for LIP05 were octanoic acid and decanoic acid, while the mutants I30K and I30D showed substrate preference toward pentanoic acid, the enzyme activity of mutants I30K and I30D was 113.66 and 109.39% relative to that of LIP05, but the enzyme activity of other mutants was reduced or even completely lost (Figure 3A). The substrate spectrum of mutants I30K and I30D was changed compared with LIP05, and the most suitable substrate was changed from long and medium chain fatty acids to short and medium chain fatty acids, and ethyl butyrate could be synthesized (Supplementary Figure S9). For the hexanoic acid, the mutant I30T was increased by 59.20% compared with LIP05, the mutant I30A, I30V, I30K, and I30D also increased slightly, while the mutant L37K showed a significant decrease (Figure 3B). For the octanoic acid, the mutants I30A, I30V, I30T and L37A showed improvement compared with LIP05, I30V and L37A were improved by 41.51 and 42.73%, respectively, and L37K showed a significant decrease (Figure 3C). For the long-chain substrate decanoic acid, I30A, I30V and I30T all showed increased enzyme activity, I30A and I30V were increased by 41.07 and 32.93% (Figure 3D). The abilities of I30A and I30V to synthesize ethyl octanoate and ethyl decanoate were both significantly increased, indicating that mutation of key amino acid residues in the lid domain affected the catalytic activity of the enzyme.

**Figure 4:**
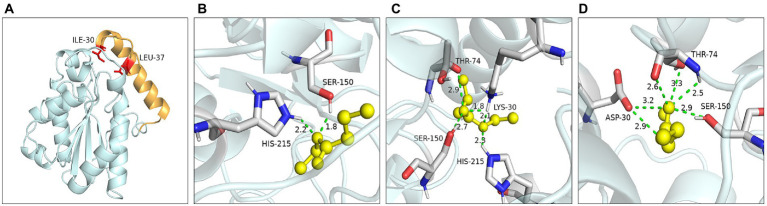
Catalytic function analysis of key residues of the lid domain. **(A)** Spatial position of Ile30 and Leu37. **(B)** Molecular docking of LIP05 to ethyl butyrate. **(C)** Molecular docking of I30K to ethyl butyrate. **(D)** Molecular docking of I30D to ethyl butyrate.

Ile30 and Leu37 were located on the lid domain of LIP05 (Figure 4A). Molecular dynamics simulation showed that the distance between Ile30 and the residues in core catalytic domain was changed with the opening of the lid, indicating that it played an important role in the change of the conformation of lid domain. The enzyme activity of mutants I30A, I30V and L37A was all increased, indicating that the steric hindrance was reduced of these mutants, making it easier for substrate-entrance and product-exit from the catalytic active pockets, thereby improving the catalytic efficiency. The mutants I30T, I30K and I30D showed changed substrate specificity, and molecular docking results indicated that the binding energies of mutants I30K and I30D to the products ethyl butyrate, ethyl pentanoate, and ethyl hexanoate were reduced (Supplementary Table S2). The hydrogen bond in mutants I30K and I30D showed longer distances than that of LIP05 (Figures 4B–D), which was beneficial to the release of the products, and generating the enhanced synthesis of short and medium chain fatty acid ethyl esters. The catalytic properties of the Ile30 and Leu37 indicated that steric hindrance affected the activity, and the change of amino acid polarity affected the substrate spectrum of the enzyme.

#### Key amino acid residues in the core catalytic domain

**Figure 5:**
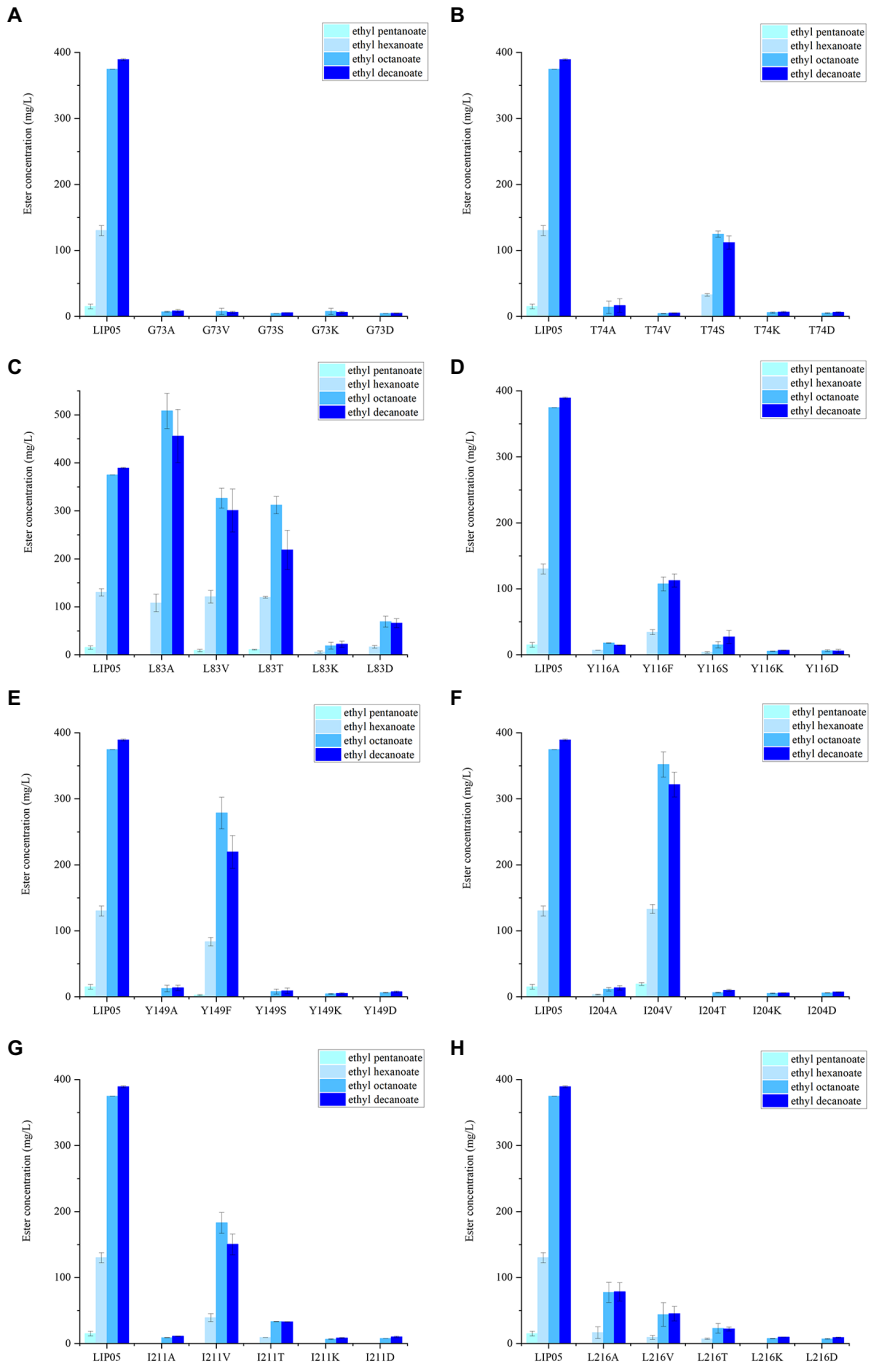
The ester synthesis ability of the mutants at **(A)** Gly73, **(B)** Thr74, **(C)** Leu83, **(D)** Tyr116, **(E)** Tyr149, **(F)** Ile204, **(G)** Ile211, **(H)** Leu216.

The catalytic active center of LIP05 in catalytic active pocket played an important role in maintaining the catalytic activity of the enzyme. The enzyme activity of mutants G73A, G73V, G73S, G73K and G73D was all completely lost (Figure 5A). The mutant T74S could maintain little enzyme activity but the other mutants at Thr74 were completely lost (Figure 5B). The enzyme activity of the mutant L83A was increased by 35.66 and 17.10% for synthesizing ethyl octanoate and ethyl decanoate, respectively (Figure 5C). For the mutants at Tyr116 and Tyr149, Y116F and Y149F could maintain a certain amount of enzyme activity, while other mutants Y116A, Y116S, Y116K, Y116D, Y149A, Y149S, Y149K, and Y149D showed almost completely lost of the activity, indicating that the mutation to the aromatic amino acid was beneficial to maintain enzyme activity (Figures 5D,E). The mutant L204V could maintain the enzyme activity, while the other mutant L204A, L204T, L204K and L204D completely lost the enzyme activity (Figure 5F). The enzyme activity of mutants at Ile211 and Leu216 was almost completely lost except the mutant I211V (Figures 5G,H).

#### Residues Gly73 and Thr74

**Figure 6:**
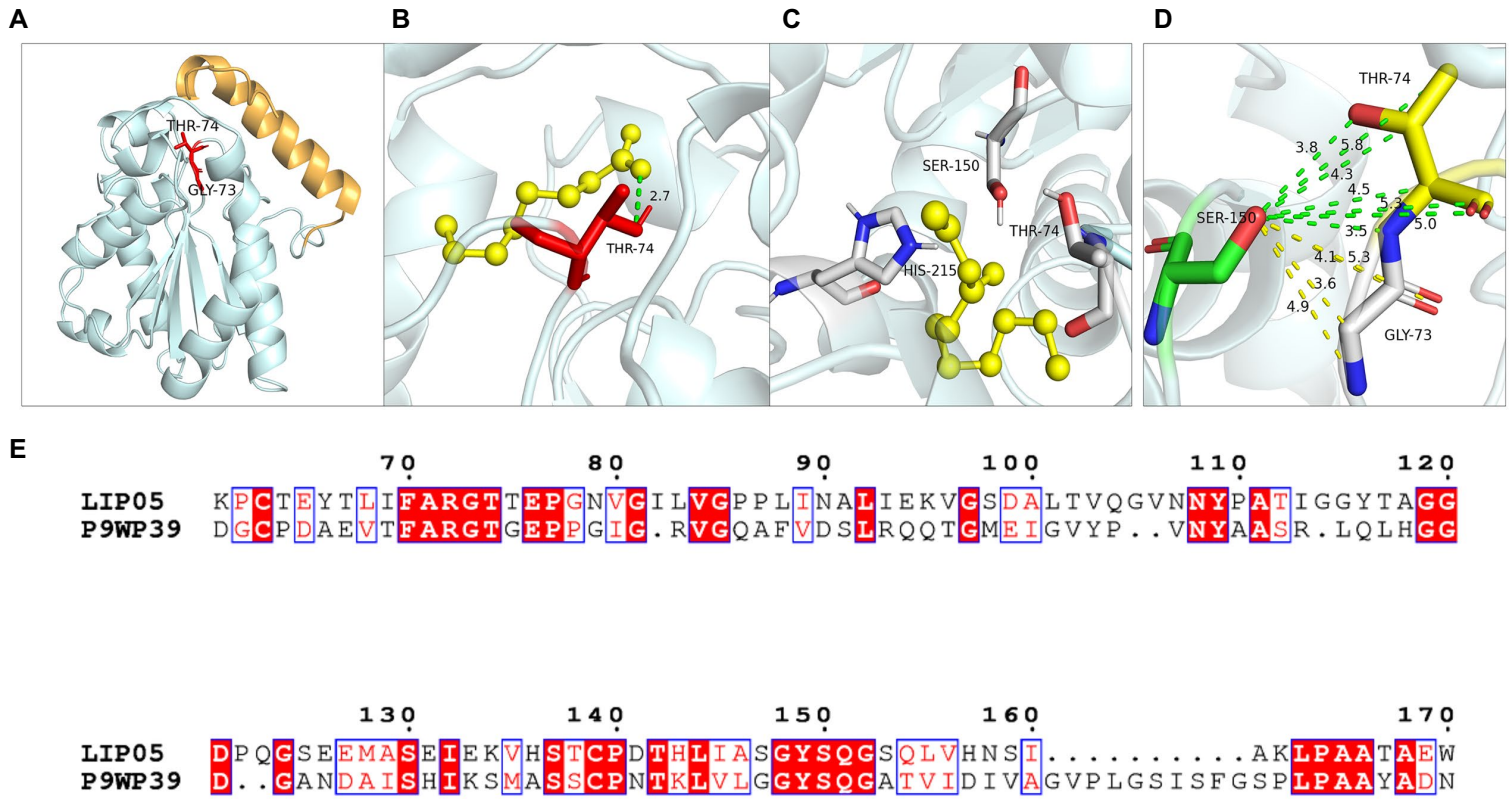
Catalytic function analysis of Gly73 and Thr74. **(A)** Spatial position of Gly73 and Thr74. **(B)** Molecular docking of LIP05 to octanoic acid at Thr74. **(C)** Molecular docking of LIP05 to ethyl octanoate at Thr74. **(D)** Oxygen anion hole at Gly73 and Thr74. **(E)** Sequence alignment of LIP05 with P9WP39.

**Figure 7:**
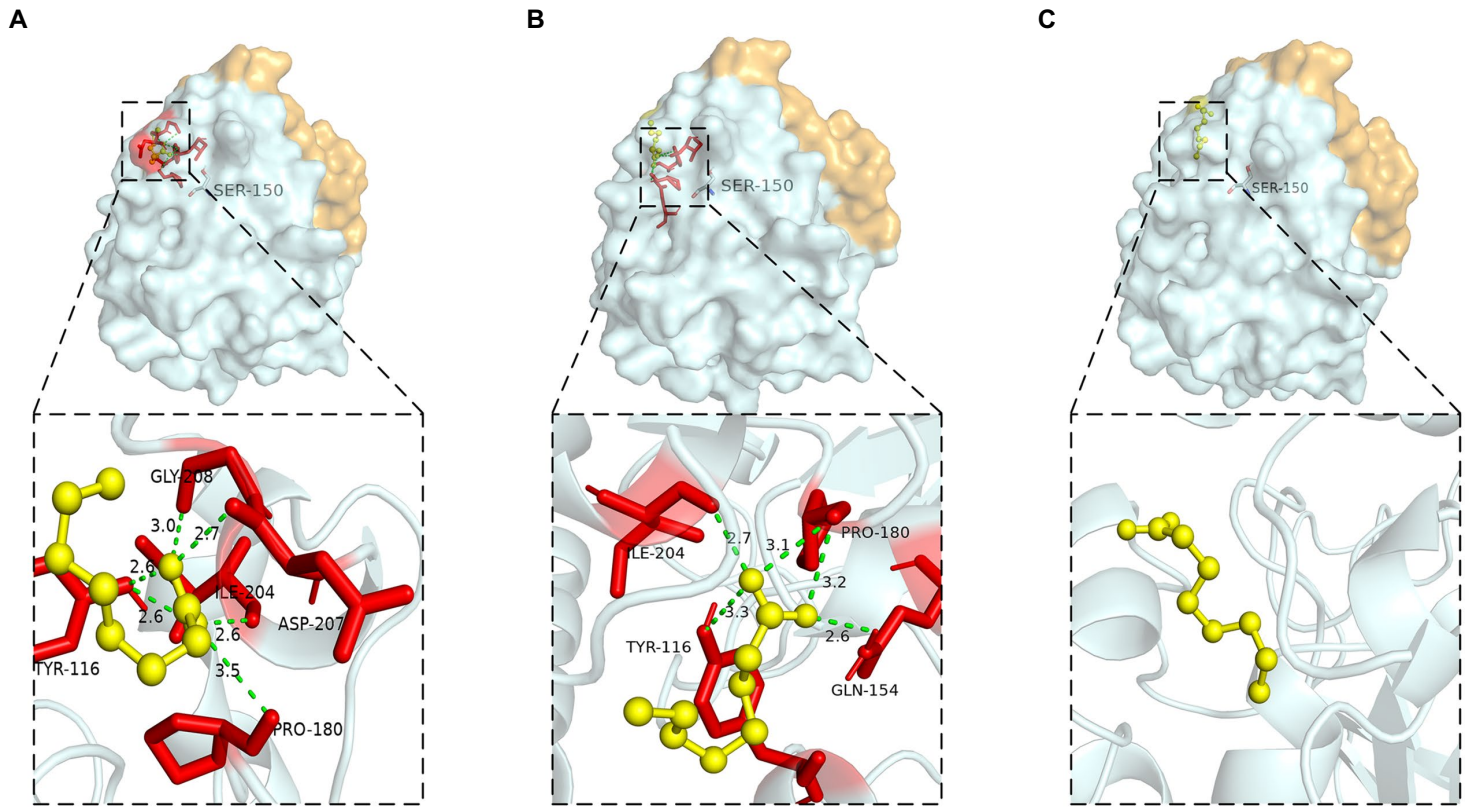
Molecular docking of **(A)** G73V, **(B)** G73K, **(C)** G73D to octanoic acid.

Hydrogen bond was a key force for maintaining the structural stability of LIP05. Both Gly73 and Thr74 were located near the catalytic active center Ser150 (Figure 6A). Hydrogen bond interactions were formed between the LIP05 and the substrate octanoic acid and the product ethyl octanoate at the Thr74 (Figures 6B,C). The distance between Thr74 and the hydroxyl of Ser150 was 2.30 Å – 5.30 Å, and the distance between Gly73 and the hydroxyl of Ser150 was about 3.50 Å – 5.30 Å (Figure 6D). About 3–5 Å around the hydroxyl group of serine was a groove like structure, and a pair of hydrogen atoms from the amino acids could be used as hydrogen donors. The structure was usually called “oxygen anion hole,” and was crucial to stabilize the transition complex formed during the catalytic process (Lai et al., 2023). Gly73 and Thr74 formed the oxyanion hole of LIP05 and belonged to the G (X) type oxyanion hole (Infanzón et al., 2018). This was similar to a cutinase derived from *Mycobacterium tuberculosis* (NCBI accession number P9WP39) that had an oxyanion hole sequence of “FARGTGE,” where “T” was the residue that constituted the oxyanion hole of the enzyme (Figure 6E; Kelkar et al., 2011). The enzyme activity of mutants was all lost at Gly73 and Thr74 because mutation destroyed the oxyanion hole, and the stability of the tetrahedral transition state during catalysis was greatly affected. The enzyme MAS1 showed similarity activity after mutating residue Phe39 next to the oxyanion hole since the destruction of the hydrogen bond affecting the affinity attack of Ser carboxyl group to the carbonyl carbon atom of the substrate, resulting in the loss of enzyme activity (Zhao et al., 2018). Furthermore, the substrate formed hydrogen bond interaction with the C-terminal amino acid residues of the mutants G73V, G73K and was completely blocked at the entrance of the channel (Figures 7A,B). For the mutant G73D, substrate was also blocked at the entrance of the channel (Figure 7C). This indicated that the mutation at Gly73 and Thr74 destroyed the binding of the substrate to the active center, resulting in loss of enzyme activity.

#### Residues Leu83, Tyr116 and Tyr149

The steric hindrance and hydrophobic interaction of the residues Leu83, Tyr116 and Tyr149 played an important role in LIP05 catalysis. Leu83 was located on the loop connecting β1 and α2. The substrate binding pocket was composed of residues Leu37, Glu41, Gly73, Thr74, Thr75, Glu76, Leu83, Ile113, Tyr116, Tyr149, Ser150, Asp209, Ile211, His215, and Leu216, which was similar to the binding pocket of the enzyme 4PSC, the residues that formed this pocket were much hydrophilic than those of most other lipase pockets. The enzyme activity of the mutant L83A was increased, and this result was similar to that lipase PEL (Tang et al., 2014), Ile75, Val72, and Phe71 were the amino acid residues at substrate binding pocket of PEL, it was found that the enzyme activity of mutants I75A, V72A, and F71A were significantly improved, indicating that the mutation to alanine reduced the steric hindrance and beneficial to the improvement of enzyme activity.

**Figure 8:**
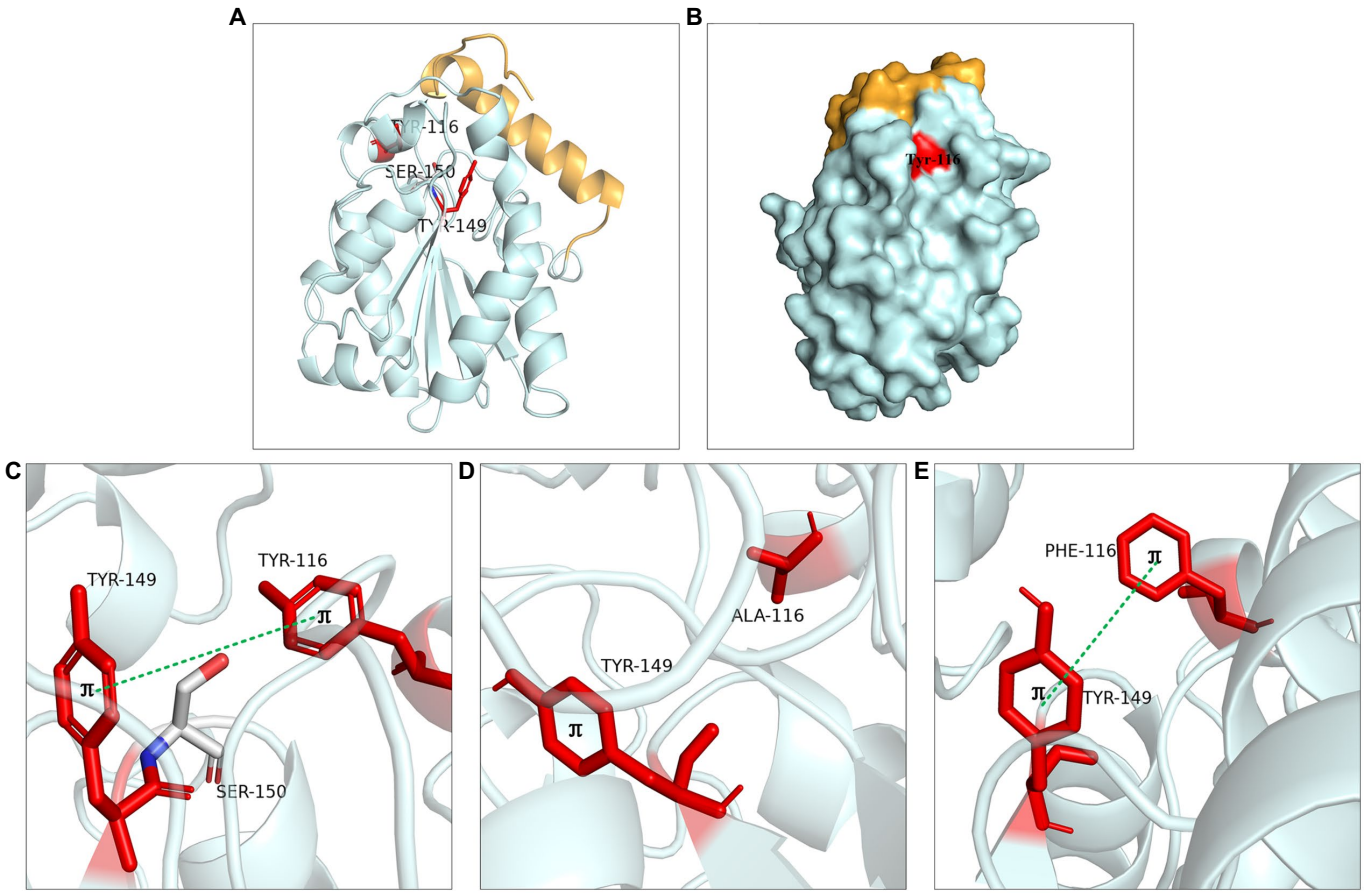
Catalytic function analysis of Tyr116 and Tyr149. **(A)** Spatial position of Tyr116 and Tyr149. **(B)** Surface model of Tyr116. **(C)** π bond interaction between Tyr116 and Tyr149. **(D)** No π bond interaction between Ala116 and Tyr149 of Y116A, **(E)** π bond interaction between the Phe116 and Tyr149 of Y116F.

The aromatic structure at Tyr116 and Tyr149 played an important role in enzyme activity. These two residues were located near the catalytic active center (Figure 8A). Tyr116 was exposed on the protein surface and was well contacted with the solvent (Figure 8B). Molecular dynamics simulation showed that Tyr116 played an important role in controlling opening substrate channels. The π-bond stacking was formed between residues Tyr116 and Tyr149, it was more likely to serve as a stabilizing force for the active site cavity (Figure 8C). The enzyme activity of mutants at Tyr116 and Tyr149 was lost because that π bond stacking could not formed between Tyr149 and Ala116 when Tyr116 was mutated to alanine (Figure 8D). However, the enzyme activity could be detected at Y116F and Y149F because the π-bond stacking between Tyr149 and Phe116 could be still formed (Figure 8E). The results were similar to the lipase SMG1, although the mutants W229A and W229L reduced the steric hindrance and maintained the hydrophobicity of the alkyl chain-binding cleft, the enzyme activity was reduced. However, the enzyme activity of mutant W229F was not decreased significantly because the aromatic structure was maintained. This proved that maintaining aromatic structure at this position was favorable for keeping the activity of the enzyme (Gao et al., 2014).

#### Residues Ile204, Ile211 and Leu216

**Figure 9:**
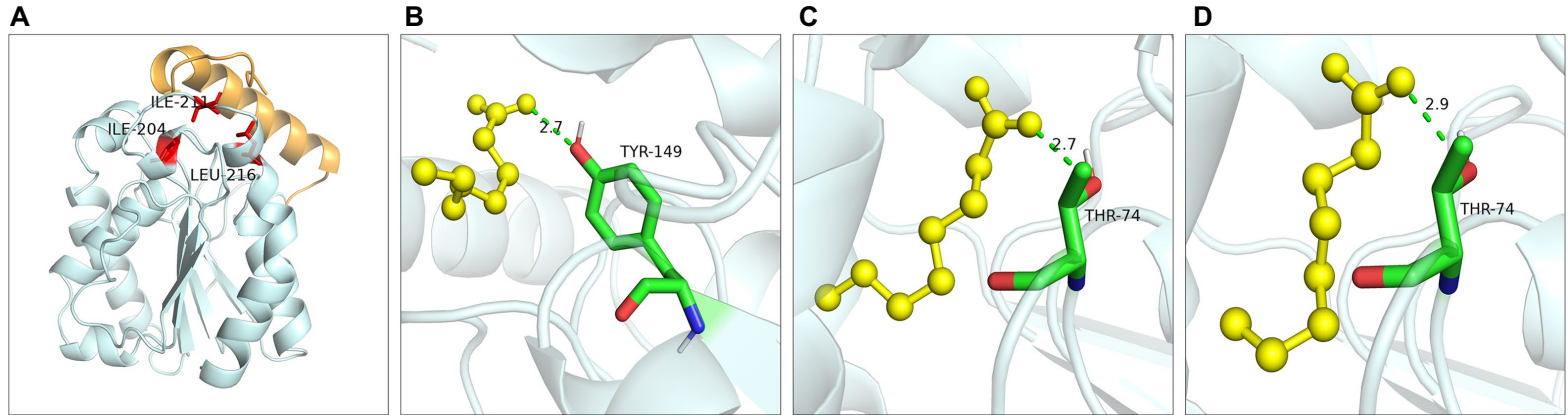
Catalytic function analysis of Ile204, Ile211 and Leu216. **(A)** Spatial position of Ile204, Ile211 and Leu216. Molecular docking of **(B)** L216T, **(C)** L216K, **(D)** L216D to octanoic acid.

Hydrophobic interaction played crucial function of these residues. Ile204 was located on a small α-helix α7, and Ile211 was located on the loop between α7 and α8 (Figure 9A). Hydrophobic interactions were formed between Ile204, Ile211, Leu216 and substrates. With the reduction of the hydrophobic interaction of the mutants, the enzyme activity was significantly reduced or even completely lost, since the reduction of hydrophobicity was not conducive to the stable binding of the substrate, resulting in the loss of enzyme activity. Leu216 was located on the α-helix α8 of the C-terminal, which was exposed on the surface of the enzyme and was close to the residue His215. Molecular docking results showed that the substrate could not form hydrogen bonds with the Ser150 and His215 in the mutants L216T, L216K, and L216D (Figures 9B–D), indicating that the hydrogen bond was destroyed and substrate could not bind to the active center after mutation.

#### The proposed catalytic mechanism of LIP05

**Figure 10:**
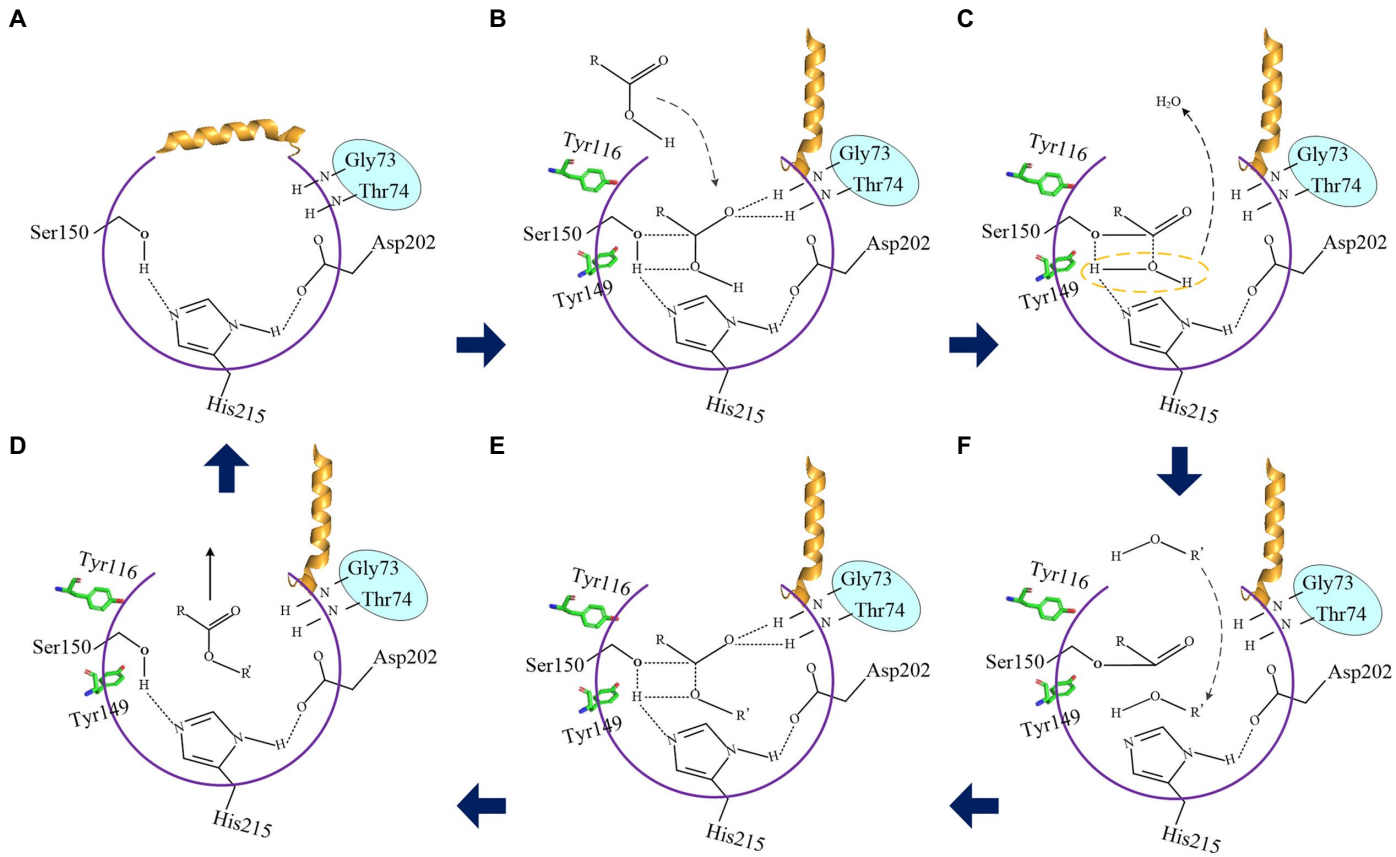
The proposed catalytic mechanism of LIP05. **(A)** Closed state of the lid domain of LIP05. **(B)** Lid domain of LIP05 opened, and the substrate acid entered into the catalytic active center. **(C)** Tetrahedral transition state 1. **(D)** Substrate alcohol entered into the catalytic active center. **(E)** Tetrahedral transition state 2. **(F)** The esterification reaction was completed.

The esterification reaction of α/β fold hydrolase was a reversible process (Stergiou et al., 2013). Results indicated that the reduction of steric hindrance and the change of amino acid polarity of residues Ile30 and Leu37 on the lid domain of LIP05 played an important role in the improvement of enzyme activity and the change of substrate spectrum (Figure 3). When the lid domain of LIP05 was opened, substrates could be allowed to entrance into the catalytic active pocket in an aqueous phase through a substrate entrance channel with a width of 3.70 Å formed by the change in spatial distance of Tyr116 and Ile204 (Figures 10A,B). The side chain hydroxyl oxygen atom of Ser150 nucleophilically attacked the carboxyl carbon atom of the substrate. The electron was transferred to the carboxyl oxygen atom of the substrate to generate an oxygen anion, and formed hydrogen bonds with the hydrogen atom of the oxygen anion hole residues Gly73 and Thr74 to maintain the stability of enzyme-substrate complex (Figure 10B). Meanwhile, the π-bond stacking was formed between the two residues Tyr116 and Tyr149, and making the catalytic active center more stable. Residues with a distance of about 4 Å from the enzyme could form hydrophobic interactions with the substrate, including Leu83, Ile204, Ile113, Ile211, and Leu216 (Supplementary Figure S6). These residues participated in the formation of the hydrophobic substrate binding pocket. After the substrate was stably bound to the LIP05, a covalent bond was formed between the carbon atom, substrate and the oxygen atom of the residue Ser150, forming a tetrahedral transition state 1 (Figures 10C,D). Thereafter, a water molecule was removed to form acyl-Ser150. The alcohol entered into the catalytic active center and attacked the carbonyl carbon atom of the acyl-Ser150, forming a tetrahedral transition state 2 (Figure 10E). Finally, the carbon atom of tetrahedral transition state 2 formed a covalent bond with the alcohol oxygen atom, and the covalent bond between the side chain hydroxyl oxygen atom of Ser150 and the carbon atom of substrate was broken to release the fatty acid ethyl esters (Figure 10F).

## Conclusion

Fatty acid ethyl esters play an important role for the flavor of strong-flavor Baijiu. In this study, the structure of LIP05 and the key sites at lid domain and core catalytic domain were analyzed, and the molecular mechanism of LIP05 catalyzing fatty acid ethyl esters synthesis in aqueous phase was proposed. To the best of our knowledge, this work revealed the enzymatic mechanism for flavor ester synthesis under aqueous phase from baijiu microorganism for the first time. This study provided a reference for identifying and developing other enzymes with ester synthases properties in aqueous systems from baijiu microorganisms and will promote the scientific understanding of ester synthesis in baijiu fermentation process.

## Data availability statement

The original contributions presented in the study are included in the article/Supplementary material, further inquiries can be directed to the corresponding author.

## Author contributions

JZ: methodology, investigation, data curation, and writing— original draft. YX: methodology, investigation, data curation, funding acquisition, and writing—original draft. HL and JZ: data curation and methodology. DZ: methodology and investigation. ML, XL, ZD, WD, MY, WL, and CZ: data curation. BS: review and editing, and funding acquisition. XL: review and editing, funding acquisition, and supervision. All authors contributed to the article and approved the submitted version.

## Funding

This work was supported by National Natural Science Foundation of China (31830069, 32072165, and 31801467), Beijing Municipal Natural Science Foundation and Beijing Municipal Education Commission (KM202110011003 and KZ202010011018), Key Laboratory of Wuliangye flavor Liquor Solid-state Fermentation, China National Light Industry (2021JJ006).

## Conflict of interest

DZ and JZ were employed by the company Wuliangye Yibin Co., Ltd.

The remaining authors declare that the research was conducted in the absence of any commercial or financial relationships that could be construed as a potential conflict of interest.

## Publisher’s note

All claims expressed in this article are solely those of the authors and do not necessarily represent those of their affiliated organizations, or those of the publisher, the editors and the reviewers. Any product that may be evaluated in this article, or claim that may be made by its manufacturer, is not guaranteed or endorsed by the publisher.

## Footnotes

^1^ https://zinc.docking.org/

^2^ https://www.gromacs.org/

^3^ https://github.com/openmm/pdbfixer

^4^ https://www.genome.jp/tools-bin/clustalw

^5^ https://espript.ibcp.fr/ESPript/cgi-bin/ESPript.cgi
