# Supplementary material for: Molecular mechanism of LIP05 derived from Monascus purpureus YJX-8 for synthesizing fatty acid ethyl esters under aqueous phase
Source: Front Microbiol. 2023 Jan 12;13:1107104. doi: 10.3389/fmicb.2022.1107104 (PMC9877431; doi:10.3389/fmicb.2022.1107104)
Supplement: Supplementary file 1 [file Data_Sheet_1.docx]

Supplementary information for

**Molecular mechanism of LIP05 derived from Monascus purpureus YJX-8 for synthesizing fatty acid ethyl esters under aqueous phase**

Jingrong Zhao^1,3,#^, Youqiang Xu^1,3,#^_,_ Hongyun Lu^1,3,#^, Dong Zhao^2^, Jia Zheng^2^, Mengwei Lin^1,3^, Xin Liang^1,3^, Ze Ding^1,4^, Wenqi Dong^1,4^, Maochen Yang^4^, Weiwei Li^1,3^, Chengnan Zhang^1,3^, Baoguo Sun^1,3^, Xiuting Li^1,3,4*^

^1^ Key Laboratory of Brewing Microbiome and Enzymatic Molecular Engineering, China General Chamber of Commerce, Beijing Technology and Business University, Beijing 100048, China.

^2^ Wuliangye Yibin Co., Ltd. Yibin, Sichuan 644000, China.

^3^ Beijing Advanced Innovation Center for Food Nutrition and Human Health, Beijing Technology and Business University, Beijing 100048, China.

^4^ School of Food and Health, Beijing Technology and Business University (BTBU), Beijing 100048, China.

# These authors contribute equally to this work

* Correspondence:

X. Li, Beijing Technology & Business University. No. 33, Fucheng Road, Haidian District, Beijing 100048, China, E-mail: lixt@btbu.edu.cn

**Table S1.** Primers used in this work.

| Primer name | Sequence (5’ -3’) |
| --- | --- |
| pCold-TF.F | CTGCAGTCTAGATAGGTAATC |
| pCold-TF.R | ACCACCACTACCTCGTGGCA |
| LIP05.F | TGCCACGAGGTAGTGGTGGTTCCCTGCCGCTGACGCCG |
| LIP05.R | GATTACCTATCTAGACTGCAGCGATGAAGCAGCAGACAC |
| S150A.F | CTCATCGCATCAGGATACTCGCAGGGTTCGCAGCTCGTT |
| S150A.R | TGCGAGTATCCTGATGCGATGAG |
| D202A.F | ACGGCTTGCCATGATGGCGATAATATCTGCCAGGATGGG |
| D202A.R | TTATCGCCATCATGGCAAGCCGT |
| H215A.F | GATCTGATCTTGCCTGCACATTTGACATATGCGGAGAAT |
| H215A.R | AAATGTGCAGGCAAGATCAGATC |
| I30A.F | CCCGCCATAAACGGCACCGCAAATGCTGTCGTCGGCATC |
| I30A.R | TTTGCGGTGCCGTTTATGGCGGG |
| I30V.F | CCCGCCATAAACGGCACCGTGAATGCTGTCGTCGGCATC |
| I30V.R | TTCACGGTGCCGTTTATGGCGGG |
| I30T.F | CCCGCCATAAACGGCACCACCAATGCTGTCGTCGGCATC |
| I30T.R | TTGGTGGTGCCGTTTATGGCGGG |
| I30K.F | CCCGCCATAAACGGCACCAAAAATGCTGTCGTCGGCATC |
| I30K.R | TTTTTGGTGCCGTTTATGGCGGG |
| I30D.F | CCCGCCATAAACGGCACCGATAATGCTGTCGTCGGCATC |
| I30D.R | TTATCGGTGCCGTTTATGGCGGG |
| L37A.F | ATGCTGTCGTCGGCATCGCAACCGATTTCGAGACACTC |
| L37A.R | GTTGCGATGCCGACGACAGCAT |
| L37V.F | ATGCTGTCGTCGGCATCGTGACCGATTTCGAGACACTC |
| L37V.R | GTCACGATGCCGACGACAGCAT |
| L37T.F | ATGCTGTCGTCGGCATCACCACCGATTTCGAGACACTC |
| L37T.R | GTGGTGATGCCGACGACAGCAT |
| L37K.F | ATGCTGTCGTCGGCATCAAAACCGATTTCGAGACACTC |
| L37K.R | GTTTTGATGCCGACGACAGCAT |
| L37D.F | ATGCTGTCGTCGGCATCGATACCGATTTCGAGACACTC |
| L37D.R | GTATCGATGCCGACGACAGCAT |
| G73A.F | ACCCTCATTTTTGCACGGGCAACCACCGAACCTGGCAAT |
| G73A.R | GTTGCCCGTGCAAAAATGAGGGT |
| G73V.F | ACCCTCATTTTTGCACGGGTGACCACCGAACCTGGCAAT |
| G73V.R | GTCACCCGTGCAAAAATGAGGGT |
| G73S.F | ACCCTCATTTTTGCACGGAGCACCACCGAACCTGGCAAT |
| G73S.R | GTGCTCCGTGCAAAAATGAGGGT |
| G73K.F | ACCCTCATTTTTGCACGGAAAACCACCGAACCTGGCAAT |
| G73K.R | GTTTTCCGTGCAAAAATGAGGGT |
| G73D.F | ACCCTCATTTTTGCACGGGATACCACCGAACCTGGCAAT |
| G73D.R | GTATCCCGTGCAAAAATGAGGGT |
| T74A.F | CTCATTTTTGCACGGGGGGCAACCGAACCTGGCAATGTC |
| T74A.R | GTTGCCCCCCGTGCAAAAATGAG |
| T74V.F | CTCATTTTTGCACGGGGGGTGACCGAACCTGGCAATGTC |
| T74V.R | GTCACCCCCCGTGCAAAAATGAG |
| T74S.F | CTCATTTTTGCACGGGGGAGCACCGAACCTGGCAATGTC |
| T74S.R | GTGCTCCCCCGTGCAAAAATGAG |
| T74K.F | CTCATTTTTGCACGGGGGAAAACCGAACCTGGCAATGTC |
| T74K.R | GTTTTCCCCCGTGCAAAAATGAG |
| T74D.F | CTCATTTTTGCACGGGGGGATACCGAACCTGGCAATGTC |
| T74D.R | GTATCCCCCCGTGCAAAAATGAG |
| L83A.F | CCTGGCAATGTCGGCATCGCAGTTGGACCCCCGCTGATC |
| L83A.R | ACTGCGATGCCGACATTGCCAGG |
| L83V.F | CCTGGCAATGTCGGCATCGTGGTTGGACCCCCGCTGATC |
| L83V.R | ACCACGATGCCGACATTGCCAGG |
| L83T.F | CCTGGCAATGTCGGCATCACCGTTGGACCCCCGCTGATC |
| L83T.R | ACGGTGATGCCGACATTGCCAGG |
| L83K.F | CCTGGCAATGTCGGCATCAAAGTTGGACCCCCGCTGATC |
| L83K.R | ACTTTGATGCCGACATTGCCAGG |
| L83D.F | CCTGGCAATGTCGGCATCGATGTTGGACCCCCGCTGATC |
| L83D.R | ACATCGATGCCGACATTGCCAGG |
| Y116A.F | CCTGCCACTATTGGGGGGTTTACGGCTGGCGGAGATCCT |
| Y116A.R | GTAAACCCCCCAATAGTGGCAGG |
| Y116F.F | CCTGCCACTATTGGGGGGTTTACGGCTGGCGGAGATCCT |
| Y116F.R | GTAAACCCCCCAATAGTGGCAGG |
| Y116S.F | CCTGCCACTATTGGGGGGAGCACGGCTGGCGGAGATCCT |
| Y116S.R | GTGCTCCCCCCAATAGTGGCAGG |
| Y116K.F | CCTGCCACTATTGGGGGGAAAACGGCTGGCGGAGATCCT |
| Y116K.R | GTTTTCCCCCCAATAGTGGCAGG |
| Y116D.F | CCTGCCACTATTGGGGGGGATACGGCTGGCGGAGATCCT |
| Y116D.R | GTATCCCCCCCAATAGTGGCAGG |
| Y149A.F | CATCTCATCGCATCAGGAGCATCGCAGGGTTCGCAGCTC |
| Y149A.R | GATGCTCCTGATGCGATGAGATG |
| Y149F.F | CATCTCATCGCATCAGGATTTTCGCAGGGTTCGCAGCTC |
| Y149F.R | GAAAATCCTGATGCGATGAGATG |
| Y149S.F | CATCTCATCGCATCAGGAAGCTCGCAGGGTTCGCAGCTC |
| Y149S.R | GAGCTTCCTGATGCGATGAGATG |
| Y149K.F | CATCTCATCGCATCAGGAAAATCGCAGGGTTCGCAGCTC |
| Y149K.R | GATTTTCCTGATGCGATGAGATG |
| Y149D.F | CATCTCATCGCATCAGGAGATTCGCAGGGTTCGCAGCTC |
| Y149D.R | GAATCTCCTGATGCGATGAGATG |
| I204A.F | TGCCATGATGGCGATAATGCATGCCAGGATGGGGATCTG |
| I204A.R | CATGCATTATCGCCATCATGGCA |
| I204V.F | TGCCATGATGGCGATAATGTGTGCCAGGATGGGGATCTG |
| I204V.R | CACACATTATCGCCATCATGGCA |
| I204T.F | TGCCATGATGGCGATAATACCTGCCAGGATGGGGATCTG |
| I204T.R | CAGGTATTATCGCCATCATGGCA |
| I204K.F | TGCCATGATGGCGATAATAAATGCCAGGATGGGGATCTG |
| I204K.R | CATTTATTATCGCCATCATGGCA |
| I204D.F | TGCCATGATGGCGATAATGATTGCCAGGATGGGGATCTG |
| I204D.R | CAATCATTATCGCCATCATGGCA |
| I211A.F | TGCCAGGATGGGGATCTGGCATTGCCTGCACATTTGACA |
| I211A.R | AATGCCAGATCCCCATCCTGGCA |
| I211V.F | TGCCAGGATGGGGATCTGGTGTTGCCTGCACATTTGACA |
| I211V.R | AACACCAGATCCCCATCCTGGCA |
| I211T.F | TGCCAGGATGGGGATCTGACCTTGCCTGCACATTTGACA |
| I211T.R | AAGGTCAGATCCCCATCCTGGCA |
| I211K.F | TGCCAGGATGGGGATCTGAAATTGCCTGCACATTTGACA |
| I211K.R | AATTTCAGATCCCCATCCTGGCA |
| I211D.F | TGCCAGGATGGGGATCTGGATTTGCCTGCACATTTGACA |
| I211D.R | AAATCCAGATCCCCATCCTGGCA |
| L216A.F | CTGATCTTGCCTGCACATGCAACATATGCGGAGAATGTG |
| L216A.R | GTTGCATGTGCAGGCAAGATCAG |
| L216V.F | CTGATCTTGCCTGCACATGTGACATATGCGGAGAATGTG |
| L216V.R | GTCACATGTGCAGGCAAGATCAG |
| L216T.F | CTGATCTTGCCTGCACATACCACATATGCGGAGAATGTG |
| L216T.R | GTGGTATGTGCAGGCAAGATCAG |
| L216K.F | CTGATCTTGCCTGCACATAAAACATATGCGGAGAATGTG |
| L216K.R | GTTTTATGTGCAGGCAAGATCAG |
| L216D.F | CTGATCTTGCCTGCACATGATACATATGCGGAGAATGTG |
| L216D.R | GTATCATGTGCAGGCAAGATCAG |

F means sense primer, R means antisense primer.

**Table S2.** The binding energies of the ligands with the receptor protein and the amino acid residues formed hydrogen bonds with the distances.

| **Enzyme** | **Parameter** | **Butyric acid** | **Ethyl butyrate** | **Pentanoic acid** | **Ethyl pentanoate** | **Hexanoic acid** | **Ethyl hexanoate** | **Octanoic acid** | **Ethyl octanoate** | **Decanoic acid** | **Ethyl decanoate** |
| --- | --- | --- | --- | --- | --- | --- | --- | --- | --- | --- | --- |
| LIP05 | Binding energy (kcal/mol) | -2.03 | -4.09 | -2.05 | -3.7 | -2.6 | -3.33 | -2.7 | 1.5 | 0.43 | 1.51 |
|  | Residue formed hydrogen bond (the distance, Å) | Thr74 (3.4)  Ser150 (2.6)  His215 (2.3) | Ser150 (1.8)  His215 (2.2) | Thr74 (3.2)  Ser150 (3.0)  His215 (2.3) | Thr74 (3.1, 2.9)  Ser150 (1.9)  His215 (2.5) | Thr74 (2.6) | Ser150 (2.6)  His215 (2.2) | Thr74 (2.7) | Thr74 (3.3)  Ser150 (2.6)  His215 (2.3) | Thr74 (2.8) | Thr74 (2.4, 2.5)  Tyr149 (3.3)  Ser150 (1.8, 3.0) |
| I30K | Binding energy (kcal/mol) | -2.31 | 0.01 | -1.52 | 1.4 | -0.52 | 1.69 | 2.37 | 9.51 | 9.46 | 24.73 |
|  | Residue formed hydrogen bond (the distance, Å) | Lys30 (2.1, 2.3)  Thr74 (2.5)  Tyr149 (2.8)  Ser150 (2.6, 2.7)  His215 (1.8) | Lys30 (1.8, 2.1)  Thr74 (2.9)  Ser150 (2.7)  His215 (2.3) | Lys30 (2.3)  Thr74 (2.5)  Tyr149 (2.8)  Ser150 (2.7, 2.7)  His215 (1.9) | Lys30 (1.9) | Lys30 (2.1, 2.3)  Thr74 (2.4)  Tyr149 (3.0)  Ser150 (2.5, 2.6)  His215 (1.8) | Lys30 (2.0) | Lys30 (1.7)  Thr74 (2.7)  Ser150 (2.7)  His215 (2.7) | Lys30 (2.1) | Lys30 (2.0)  Thr74 (2.5, 2.6)  Ser150 (2.7) | Lys30 (1.6)  Tyr149 (2.9)  Ser150 (3.4)  His215 (2.0) |
| I30D | Binding energy (kcal/mol) | -1.29 | -3.48 | -1.09 | -2.97 | -1.05 | -3.16 | -0.82 | -1.28 | 0.95 | 3.72 |
|  | Residue formed hydrogen bond (the distance, Å) | Thr74 (2.6, 2.8)  Tyr149 (3.0)  Ser150 (2.8, 2.8)  His215 (1.8) | Asp30 (2.9, 3.2)  Thr74 (2.5, 2.6, 3.3)  Ser150 (2.9) | Asp30 (3.0, 3.2)  Thr74 (2.5, 3.3)  Ser150 (2.7, 3.0)  His215 (2.2) | Asp30 (2.7)  Thr74 (2.7)  His215 (1.8) | Asp30 (3.1)  Thr74 (2.8)  Ser150 (2.7) | Thr74 (2.7)  Tyr149 (3.4)  Ser150 (2.7)  His215 (2.4) | Asp30 (2.9, 3.1)  Thr74 (2.8) | Asp30 (2.5)  Thr74 (2.3)  Ser150 (2.8, 2.8)  His215 (2.6) | Asp30 (3.0, 3.1)  Thr74 (2.6) | Asp30 (2.3)  Thr74 (2.4, 2.7, 3.4)  Ser150 (1.7) |


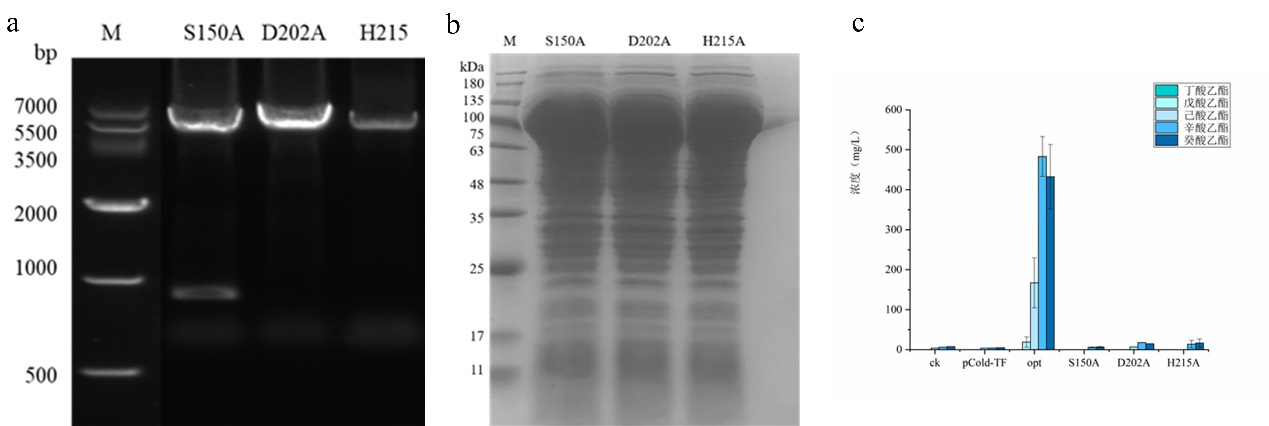


**Figure S1.** Alanine mutation of the catalytic triad.

(a) Whole plasmid PCR agarose gel electrophoresis. (b) Heterologous expression SDS-PAGE electrophoresis. (c) Ester synthesis ability determination.


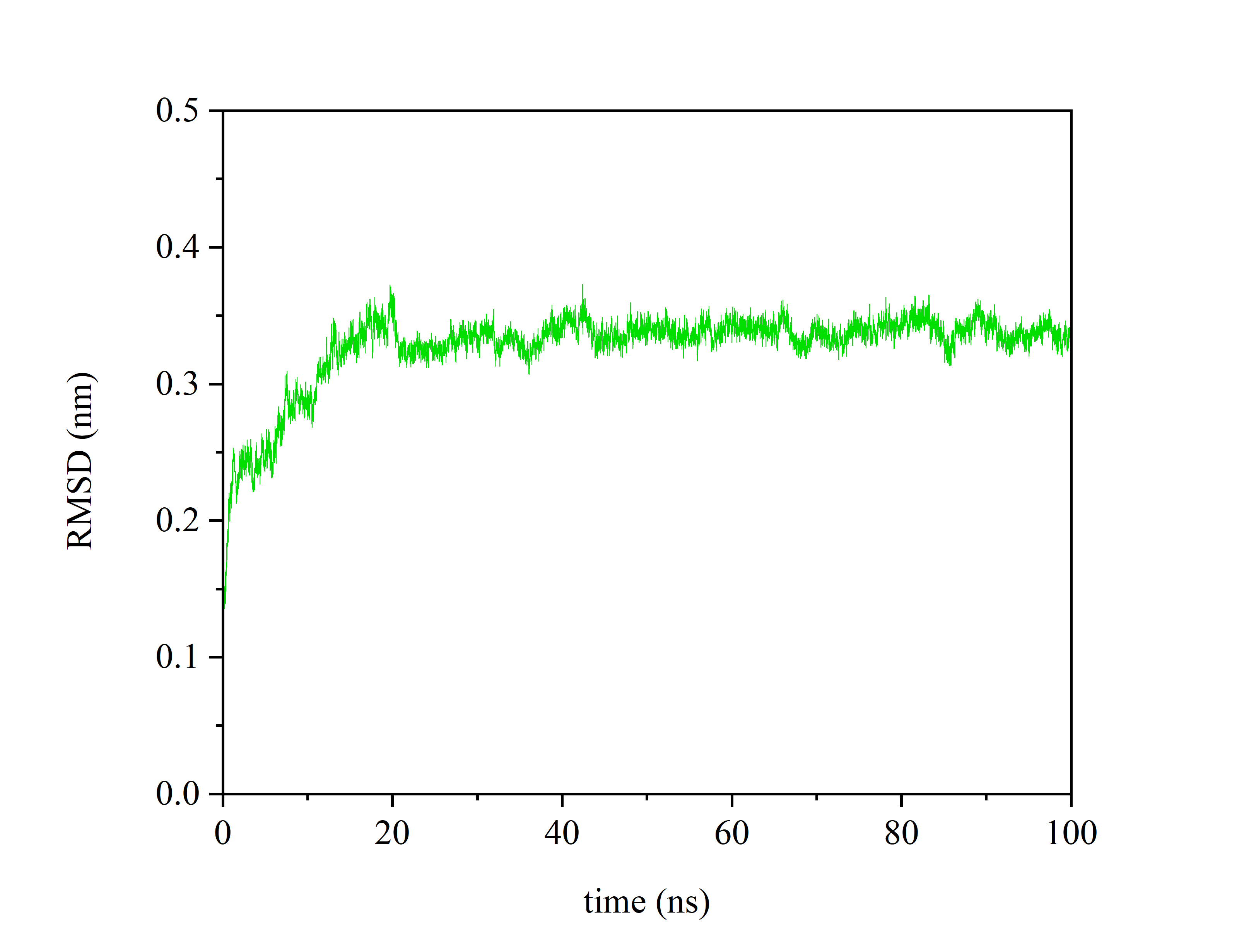


**Figure S2.**  Root Mean Square Deviation (RMSD) of LIP05.


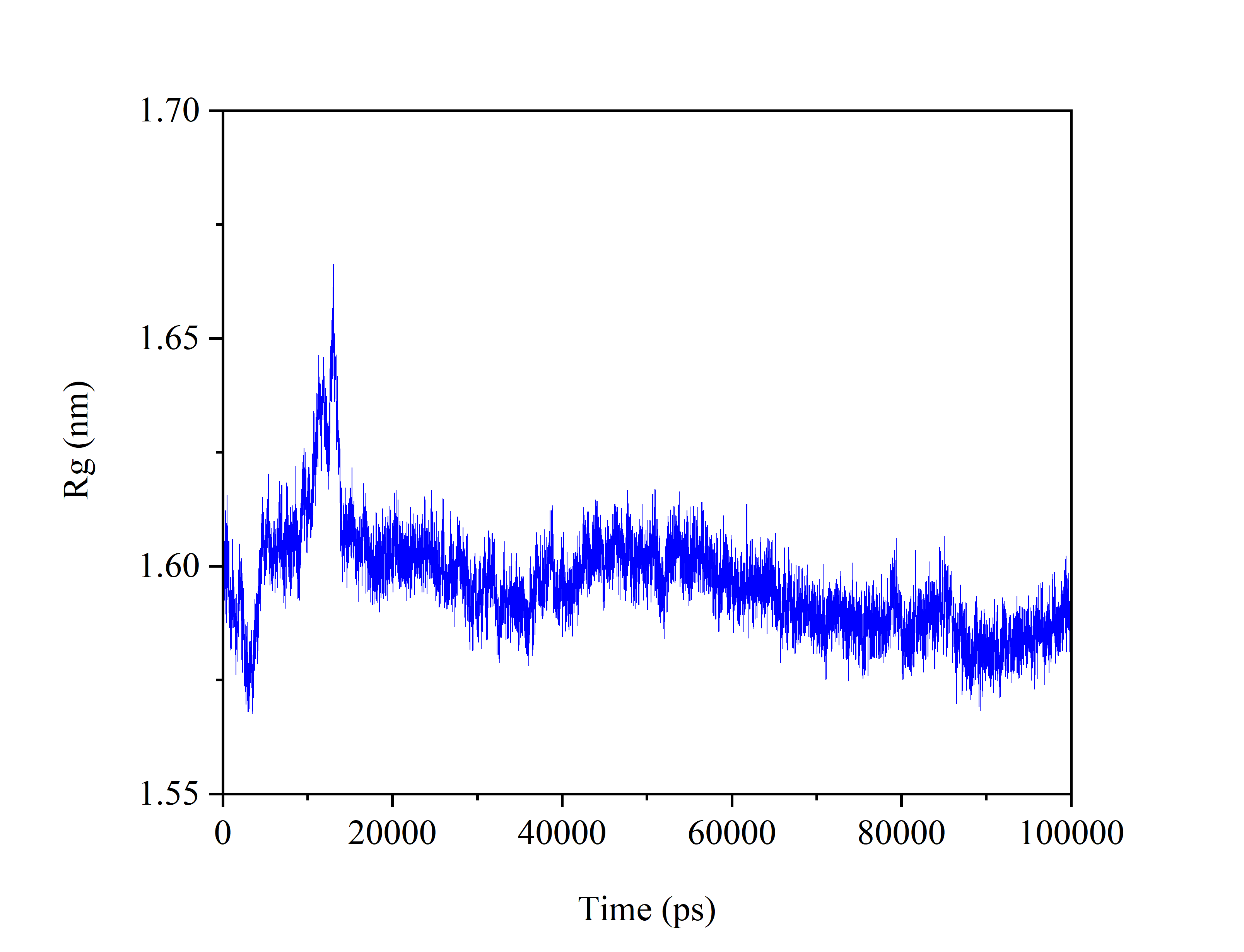


**Figure S3.**  Radius of gyration (Rg) of LIP05.


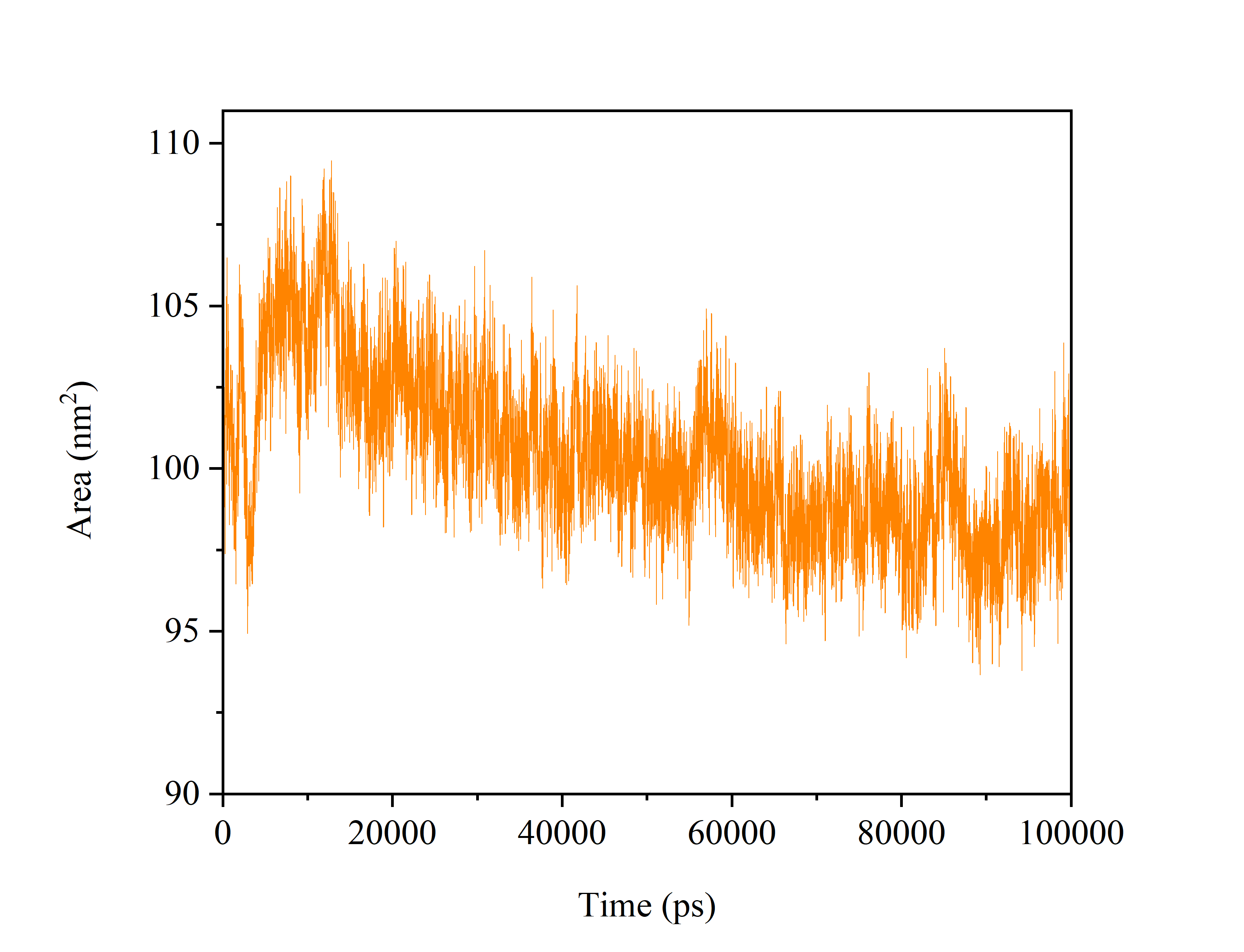


**Figure S4.** Solvent accessible surface area (SASA) of LIP05.


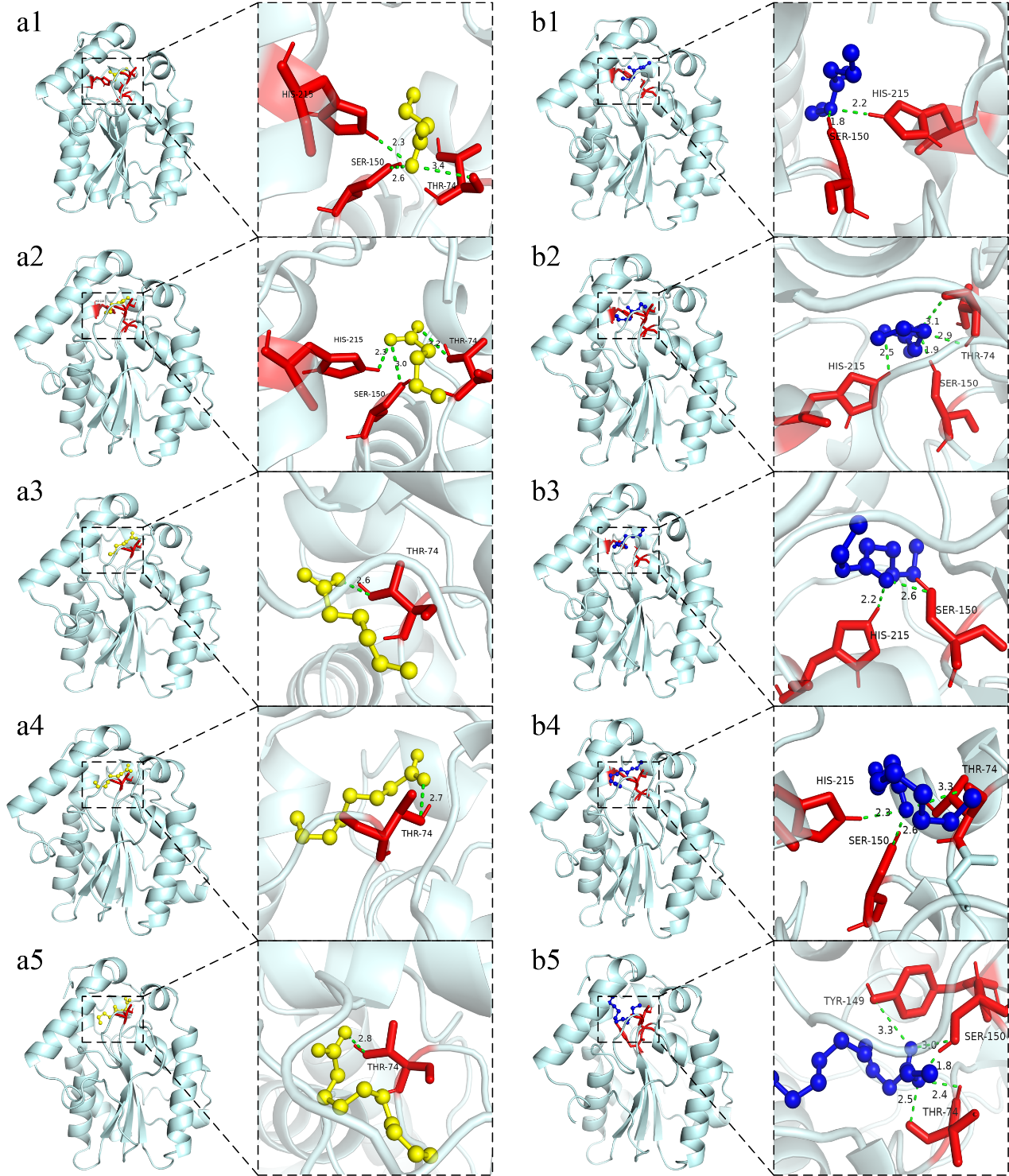


**Figure S5.** Molecular docking of LIP05.

(a1) Butyric acid. (a2) Pentanoic acid, (a3) Hexanoic acid. (a4) Octanoic acid. (a5) Decanoic acid. (b1) Ethyl butyrate. (b2) Ethyl pentanoate. (b3) Ethyl hexanoate. (b4) Ethyl octanoate. (b6) Ethyl decanoate.


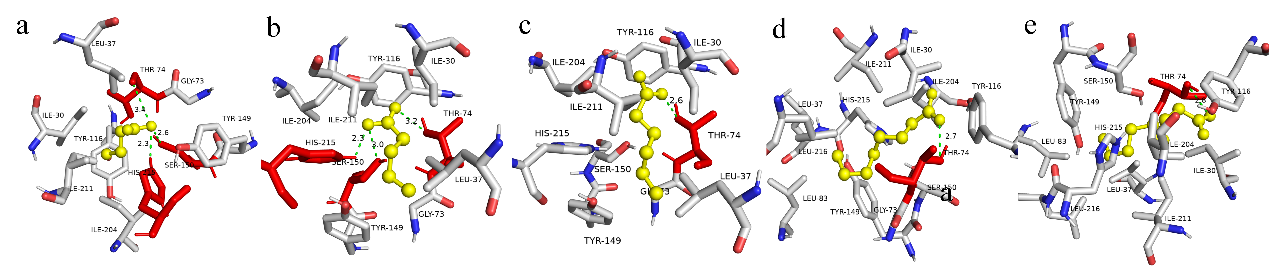


**Figure S6.** Hydrophobic interaction of LIP05 with substrates.

(a) Butyric acid. (b) Pentanoic acid. (c) Hexanoic acid. (d) Octanoic acid. (e) Decanoic acid.


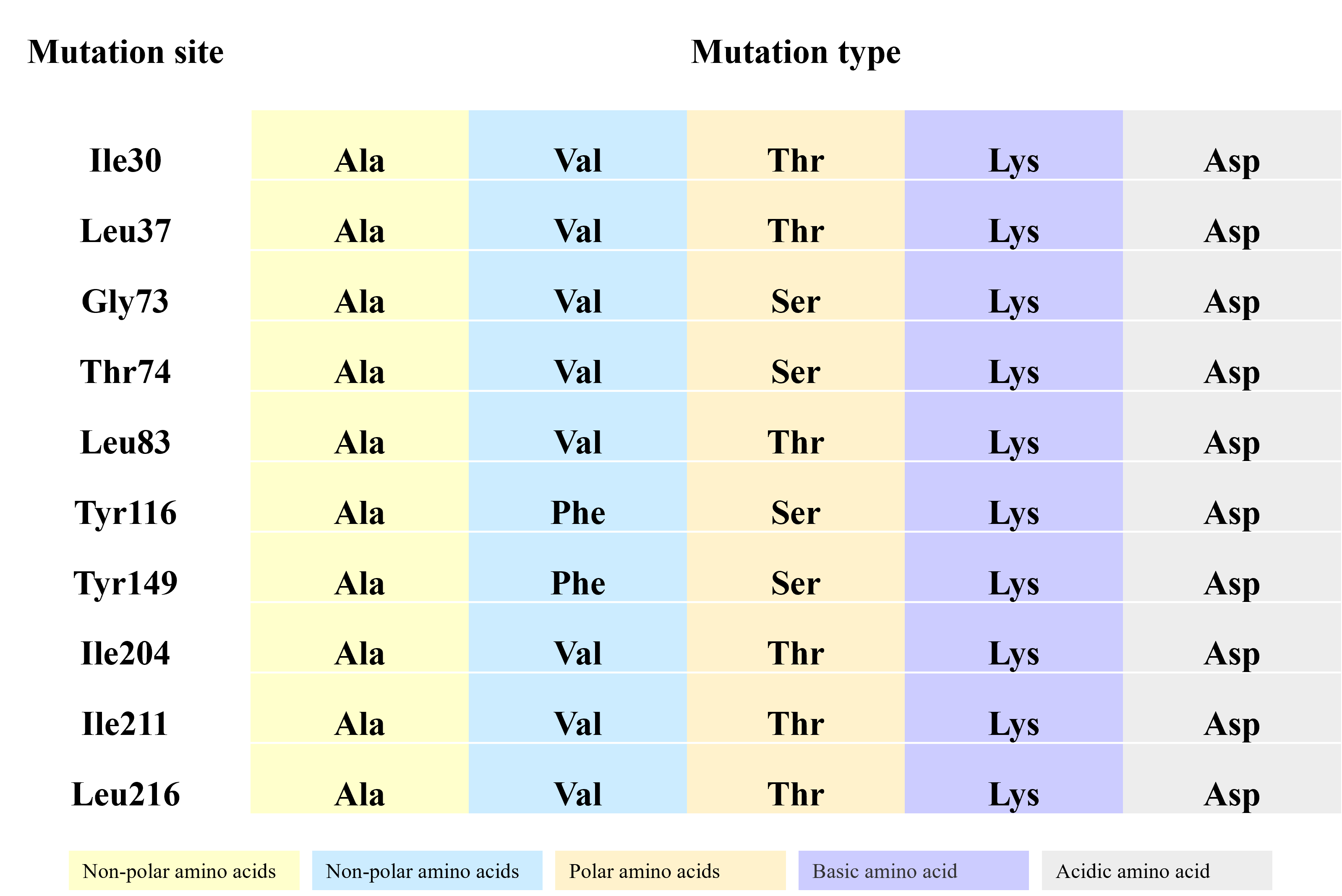


**Figure S7.** Mutation strategy of LIP05.


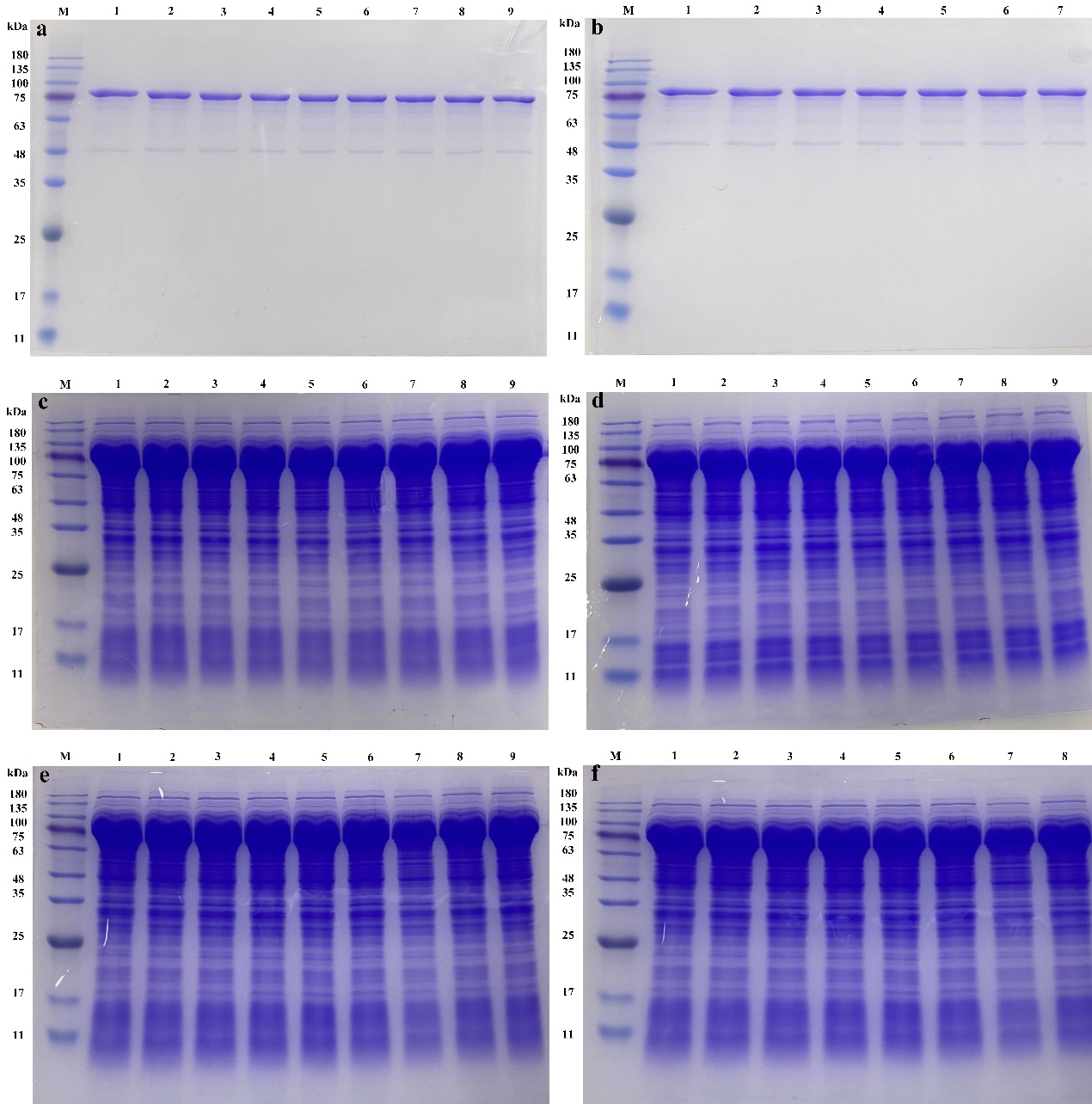


**Figure S8.** SDS-PAGE electrophoresis of heterologous expressed of LIP05 mutants.

(a) 1-9: LIP05, I30A, I30V, I30T, I30K, I30D, L37A, L37V, L37T. (b) 1-7: L37K, L37D, L83A, L83V, L83T, L83K, L83D. (c) 1-9: G73A, G73V, G73S, G73K, G73D, T74A, T74V, T74S, T74K. (d) 1-9: T74D, Y116A, Y116F, Y116S, Y116K, Y116D, Y149A, Y149F, Y149S. (e) 1-9: Y149K, Y149D, I204A, I204V, I204T, I204K, I204D, I211A, I211V. (f) 1-8: I211T, I211K, I211D, L216A, L216V, L216T, L216K, L216D.


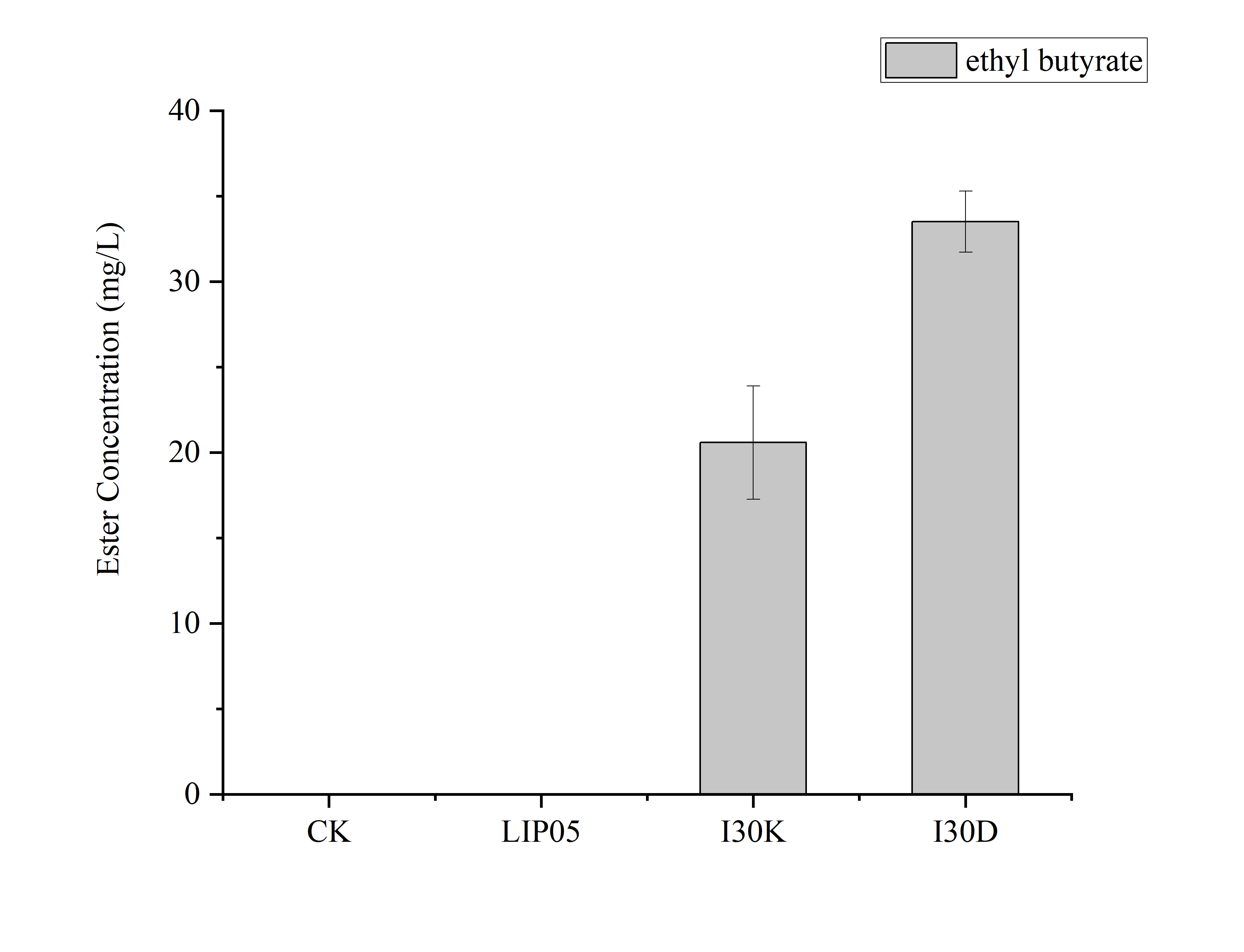


**Figure S9** The ethyl butyrate synthesis ability of the mutants I30K and I30D.
